# Mapping an Atlas of Tissue-Specific *Drosophila melanogaster* Metabolomes by High Resolution Mass Spectrometry

**DOI:** 10.1371/journal.pone.0078066

**Published:** 2013-10-29

**Authors:** Venkateswara R. Chintapalli, Mohammed Al Bratty, Dominika Korzekwa, David G. Watson, Julian A. T. Dow

**Affiliations:** 1 Strathclyde Institute for Pharmacy and Biomedical Sciences, University of Strathclyde, Glasgow, United Kingdom; 2 Institute of Molecular, Cell and Systems Biology, College of Medical, Veterinary and Life Sciences, University of Glasgow, Glasgow, United Kingdom; Imperial College London, United Kingdom

## Abstract

Metabolomics can provide exciting insights into organismal function, but most work on simple models has focussed on the whole organism metabolome, so missing the contributions of individual tissues. Comprehensive metabolite profiles for ten tissues from adult *Drosophila melanogaster* were obtained here by two chromatographic methods, a hydrophilic interaction (HILIC) method for polar metabolites and a lipid profiling method also based on HILIC, in combination with an Orbitrap Exactive instrument. Two hundred and forty two polar metabolites were putatively identified in the various tissues, and 251 lipids were observed in positive ion mode and 61 in negative ion mode. Although many metabolites were detected in all tissues, every tissue showed characteristically abundant metabolites which could be rationalised against specific tissue functions. For example, the cuticle contained high levels of glutathione, reflecting a role in oxidative defence; the alimentary canal (like vertebrate gut) had high levels of acylcarnitines for fatty acid metabolism, and the head contained high levels of ether lipids. The male accessory gland uniquely contained decarboxylated S-adenosylmethionine. These data thus both provide valuable insights into tissue function, and a reference baseline, compatible with the FlyAtlas.org transcriptomic resource, for further metabolomic analysis of this important model organism, for example in the modelling of human inborn errors of metabolism, aging or metabolic imbalances such as diabetes.

## Introduction

Metabolomics is a valuable tool for *Drosophila melanogaster* functional genomics [1]–[4]. Of the metazoans, *D. melanogaster* offers perhaps the best balance between genetic tractability, availability of well-characterised genetic mutant stocks, and organismal complexity [5], [6]. Some mutations in metabolic pathways have been studied for nearly a century [7] and interactions with, and epistatic interactions between, similar mutations, established. In previous work we have looked at the impact of the *ry*, *cho, y* and *ma-l* mutations on the *Drosophila* metabolome [1]–[3]. In addition we examined the effects of the xanthine oxidase inhibitor allopurinol on the *Drosophila* in an attempt to phenocopy the metabolic sequelae of the *ry* mutation, which we had examined earlier and observed unexpected additional effects of the drug treatment [4]. Although human metabolomics is necessarily observational, studies of simpler organisms offer the prospect of linking levels of gene expression with their impact on tissue metabolomes.

Although metabolomic approaches in this tiny animal are increasing in popularity [8]–[15], it is important to move beyond the ‘whole-organism homogenate’ approach. As a metazoan, Drosophila has multiple, functionally distinct tissues; and as the FlyAtlas.org resource has shown that gene expression can vary markedly between tissues [16], it is reasonable to predict that tissue-specific metabolomes might also differ. Few studies have addressed metabolite levels in different tissues within an organism. Carvalho *et al* determined the lipid composition of gut, fat body, wing disc salivary gland and brain in *Drosophila* larvae and found marked differences in a range of lipids between tissues [5]. In a previous study, we demonstrated differences in metabolism between head, abdomen and thorax in *Drosophila*, confirming the prediction that the production of ammonia from glutamine is greatest in the posterior tubules [6]. In the current study we have applied LC-MS platforms [17], [18] to the generation of a baseline tissue map of *Drosophila* for both polar metabolites and for a range of lipids, as an important step in establishing a comprehensive systems biology of interacting tissues within the whole organism.

## Materials and Methods

### Chemicals

Ammonium acetate, acetonitrile, methanol, propan-2-ol and chloroform were from Fisher Scientific (Leicestershire, UK), and the formic acid was from VWR (UK). All chemicals used were analytical grade. A Direct Q-3 water purification system (Millipore, Watford, UK) was used to produce the HPLC water that was used in all of the analyses.

### Extraction of Flies

Methanol: chloroform: water (3∶1∶1 v/v/v) at 0°C was used for sample quenching extraction. Ten adult (7-day) flies, with equal numbers of males and females, were collected and anesthetized on ice quickly before dissections. Then the tissues were dissected out from the flies in *Drosophila* Schneider’s medium and collected into the ice cold solvent mixture. The tissues were then homogenized for 30 s by using an ultrasonic cell disruptor (Misonix, Inc., USA). The homogenates were then centrifuged for 10 min at 4°C. Extracts were removed from the cell debris and stored at −80°C until required. Prior to the analysis, samples were kept at room temperature for 30 min. and were then placed into glass autosampler vials. Four replicates were carried out each replicate contained tissues from ten adult flies.

### Protein Determination

Fly dissections were performed as described and homogenised in 50 µl of Tris-Lysis buffer (2% (w/v) SDS, 70 mM Tris, pH 6.8) containing 2 µl of Sigma protease inhibitor cocktail (Sigma UK) in a 1.5 ml microcentrifuge tube. The sample was then centrifuged at 13 000 *g* for 10 min to remove debris and the supernatant transferred into a new tube. Bradford protein assay reagent was used to quantify the protein concentration in 1 µl samples of protein supernatant in a final volume of 50 µl (in triplicate) using Bovine Serum Albumin (BSA) protein standards. 200 µl of a 1 in 5 dilution of Bradford reagent concentrate (BioRad UK) was added to both standard and sample proteins in the wells. The absorbance at 590 nm was read using a plate reader and absorbance was plotted against the known concentration. The concentrations for protein in the tissue extracts were then quantified against the calibration curve. A multiplication factor was calculated for each tissue using whole fly as the baseline to normalise the metabolite intensities against protein content (**Supplementary Table S1**).

### LC-MS Analysis

A ZIC–HILIC column 5 µm (150×4.6 mm, HiChrom, Reading UK) was used for the analysis of polar metabolites. The sample injection volume was 10 µl, solvent A was 0.1% v/v formic acid in HPLC grade water and solvent B was 0.1% v/v formic acid in acetonitrile. A flow rate of 300 µl/min with linear gradients as follows: 80%B at (0 min) −50%B at (12 min) 50% B (12–26 min) 20%B (28 min) −20%B (28–36 min) −80% B (37 min) –80% B (37–46 min). Samples were kept in a vial tray which was set at constant temperature of 3°C to avoid any possible degradation of samples. The column was used at ambient temperature.

For lipid analysis, an ACE silica gel column (3 mm×150 mm×3 µm, HiChrom Reading U.K.) with hydrophilic interaction chromatography (HILIC) mode was used. Solvent A was 20% 2- propanol (IPA) in 20 mM ammonium formate (v/v) and solvent B was 20% IPA in acetonitrile. A flow rate of 300 µl/min was used and the injection volume was 10 µl. The gradients were linear and were as follows: 90%B at (0–5 min) −70%B at (9 min) −65%B at (13 min) −60%B at (23 min) −55%B at (28–30 min) –90%B for 31–40 min. Samples were kept in a vial tray which was set at constant temperature of 3°C to avoid any possible degradation of samples. The column was used at ambient temperature.

The LC-MS system consisted of an Accela HPLC pump interfaced with Orbitrap Exactive mass spectrometer was used in positive/negative switching mode. The instrument was calibrated according to the manufacturer’s instructions and operated at 50,000 resolution. The needle voltage was 4.5 kV in positive mode and −4.0 kV in negative ion mode, the heated capillary temperature was 320°C and the sheath and auxiliary gases 50 and 17 arbitrary units respectively. The background ions 195.0876 (+ve), 166.045 and 219.175 (-ve) were used as lock masses in order ensure the best mass calibration accuracy. The scan range was 75–1200 amu.

### Data Extraction

Firstly, data obtained from Xcalibur software were exported into Sieve Software 1.3 (Thermo Fisher Co.) where extracted ion chromatograms were aligned in 0.02 amu bins. The features obtained in Sieve were then exported into an Excel based macro written “in house” used for searching against a data base of accurate masses taken from KEGG [15], Lipidmaps [16], Human Metabolome database [17] and Metlin [18]. The lists of metabolites obtained were then carefully manually evaluated by considering the quality of the peaks and their alignment which can be viewed in Sieve. Retention times for some metabolites could be matched to the retention times of standard mixtures of metabolites (Supplementary Table S2).

## Results

### LC-MS Identifies over 500 Metabolites

We have previously described the identification of over 200 *Drosophila* metabolites using the ZIC-HILIC LC-Orbitrap MS platform; with the addition of a silica gel LC-Orbitrap MS analysis, the comprehensiveness of the analysis increases greatly. Of over 2000 features (peaks) seen reliably, 242 polar metabolites were putatively identified by exact mass across the tissues of *Drosophila*. Supplementary **Table S3** (supplementary information) shows a heat map for all the polar metabolites detected in the tissues ranked according to their abundance in the whole fly. All masses were within 1.5 ppm of the exact mass of the molecular formula suggested for a metabolite, which meant that the only competing metabolites within the database search were isomers, rather than isobaric compounds. In practice it is difficult to find known isobaric biomolecules with completely different elemental compositions which are within 5 ppm of each other [19]. Supplementary **Table S2** shows the retention times for 82 standards which were run alongside the samples.

Across the different tissues, a great variety of lipids was observed: 251 lipids in positive ion mode and 62 in negative ion mode. The lipids were putatively identified according their exact masses and are reported without specifying the nature of the acyl chains attached to the lipids; this would have required tandem MS for their elucidation. Supplementary **Table S4 and S5** in the supplementary information show heat maps of the full lipid profiles.

### Tissue Metabolomes Differ

In small model organisms, it is convenient to perform whole-organism studies; that is, to grind up and analyse the whole insect or worm, rather than to go to the painstaking effort to dissect the major tissues individually. However, the transcriptomic analysis underlying the FlyAtlas.org online expression resource showed that individual tissues had very different transcriptomes compared with whole fly. Additionally, each tissue contributed typically 5% of the whole-organism transcriptome; so even large changes in gene expression in a single tissue would be severely under-represented in the whole-organism transcriptome [16]. Does the same logic apply to metabolomes, or do the metabolomes of individual tissues resemble closely that of the whole organism?

Clear presentation of the rich data obtained from this type of study is challenging. In the figures following, we present the data as heat maps of peak areas normalised to the protein content of each tissue. In addition RSD values for the peak areas obtained for the metabolites across the four replicates have been added. The greatest sources of variability are the metabolites content of individual tissues and the success in quenching metabolism prior to manipulation. Despite these factors the RSD values for replicates of some tissues are very low. The figures have also been annotated with the level of confidence with which the metabolites have been identified. At level 1 accurate mass and matching of retention time against a standard have been obtained or in some cases abundant fragments formed in-source have increased the confidence of the ID. At level 2 the identification is based on accurate mass and in this case isomers of the metabolite are possible. The figures have been annotated with the number of alternative isomers in the databases, in many cases the isomers will be closely related. For examples methylated purines and biopterins have a number of isomeric possibilities but the isomers belong to the same class. All the lipids are identified on the basis of accurate mass and no attempt has been made to elucidate the structure of the acyl chains. The comprehensive lists of identified metabolites in each tissue are presented numerically in supplementary tables, together with relative standard deviations (RSDs); for brevity, metabolites in a particular tissue are only highlighted in the main text where their distribution pattern in the tissue is striking and statistically significant. We used a panel of 82 compounds as standards in this study; in lieu of calibration standards for every single compound discussed here, it should be emphasised that analysis is semi-quantitative in nature; however, peak area is generally accepted to provide a reasonable surrogate of abundance for particular compounds between samples. Practically it would be a large effort to quantify such a wide range of compounds and metabolomics can be divided into the discovery stage (semi-quantitative) and the targeted stage (quantitative). Thus the observations in this paper are within the discovery stage of metabolomics and thus generate hypotheses that could be investigated in a more targeted manner in the future. Furthermore, in these normalized samples, any tissue-tissue differences are likely to have functional significance. The results show that, over a range of metabolite classes, the metabolomes of individual tissues show similarities but also marked differences, as had been observed in the transcriptome [16].

### Amino Acids

**Figure 1:**
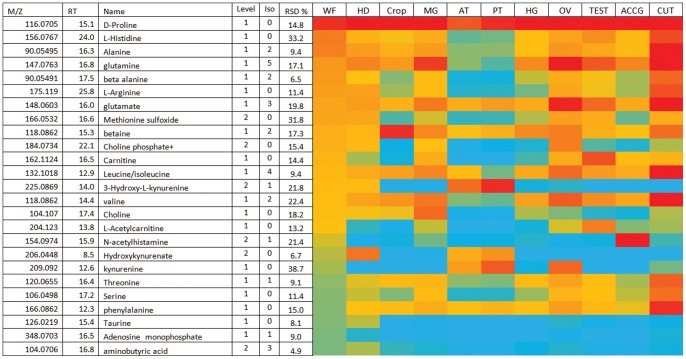
The top 25 most abundant metabolites by area response in *Drosophila* whole fly and in ten tissues from *Drosophila*. Red = area >10^8^ Yellow area >10^7^ Blue = area <10^5^.

**Fig. 1** shows the top 25 most abundant polar metabolites in the whole fly, averaged over all the tissues. It can be seen that peak area falls off rapidly (over the two orders of magnitude colour coded in the Figure) with the 25^th^ ranked metabolite in the whole fly having a peak area of *ca.* 4% of the most abundant metabolite. Proline is the most abundant metabolite, and highly uniform in peak area across the tissues, implicitly supporting the validity of normalizing samples against protein content. The amino acids dominate the top 25 list, with alanine, valine, leucine/isoleucine, phenylalanine, glutamine, glutamate, threonine, histidine, serine and taurine all included. However, it is interesting that all apart from proline show a tissue-specific pattern of expression, with (for example) consistently lower levels in the Malpighian tubules. The Malpighian tubules and hindgut serve the critical roles of generating the primary urine, and selectively reabsorbing desirable solutes, and FlyAtlas shows that *CG15088* (the fly homologue of the SLC6A19 Amino-acid resorptive transporter), is expressed almost exclusively in the tubules and hindgut. Low amino acid levels in the excretory system could thus reflect efficient recycling of these amino acids back to the blood.

### The Malpighian (Renal) Tubules are Conspicuous for Visual Pigments

**Figure 2:**
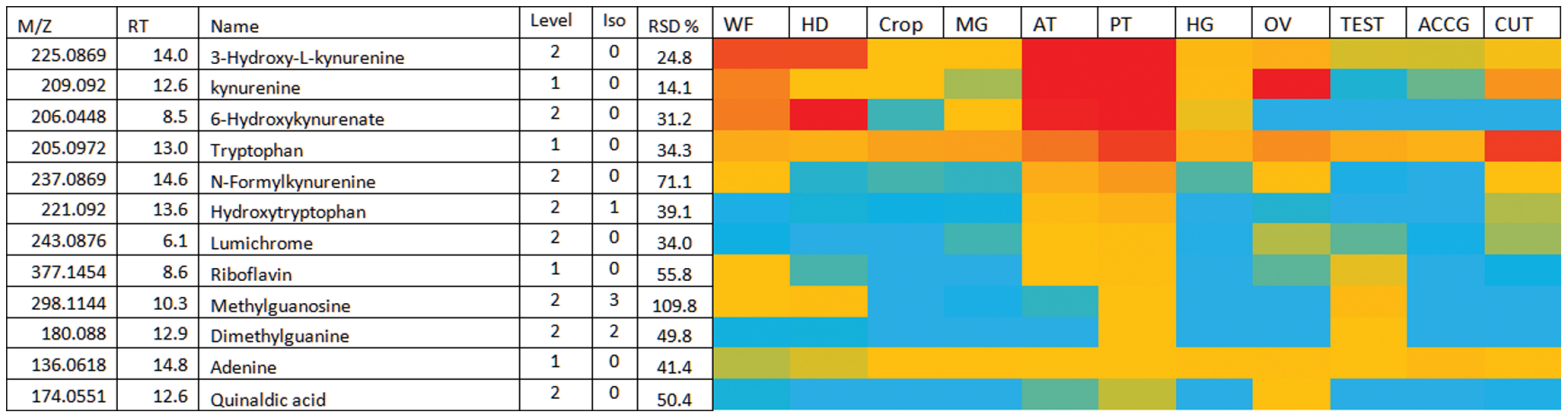
Relatively abundant metabolites in posterior tubule. Data are presented as a heat-map of peak areas, from red (>5×10^7^) to blue (<5×10^3^).

**Figure 3:**
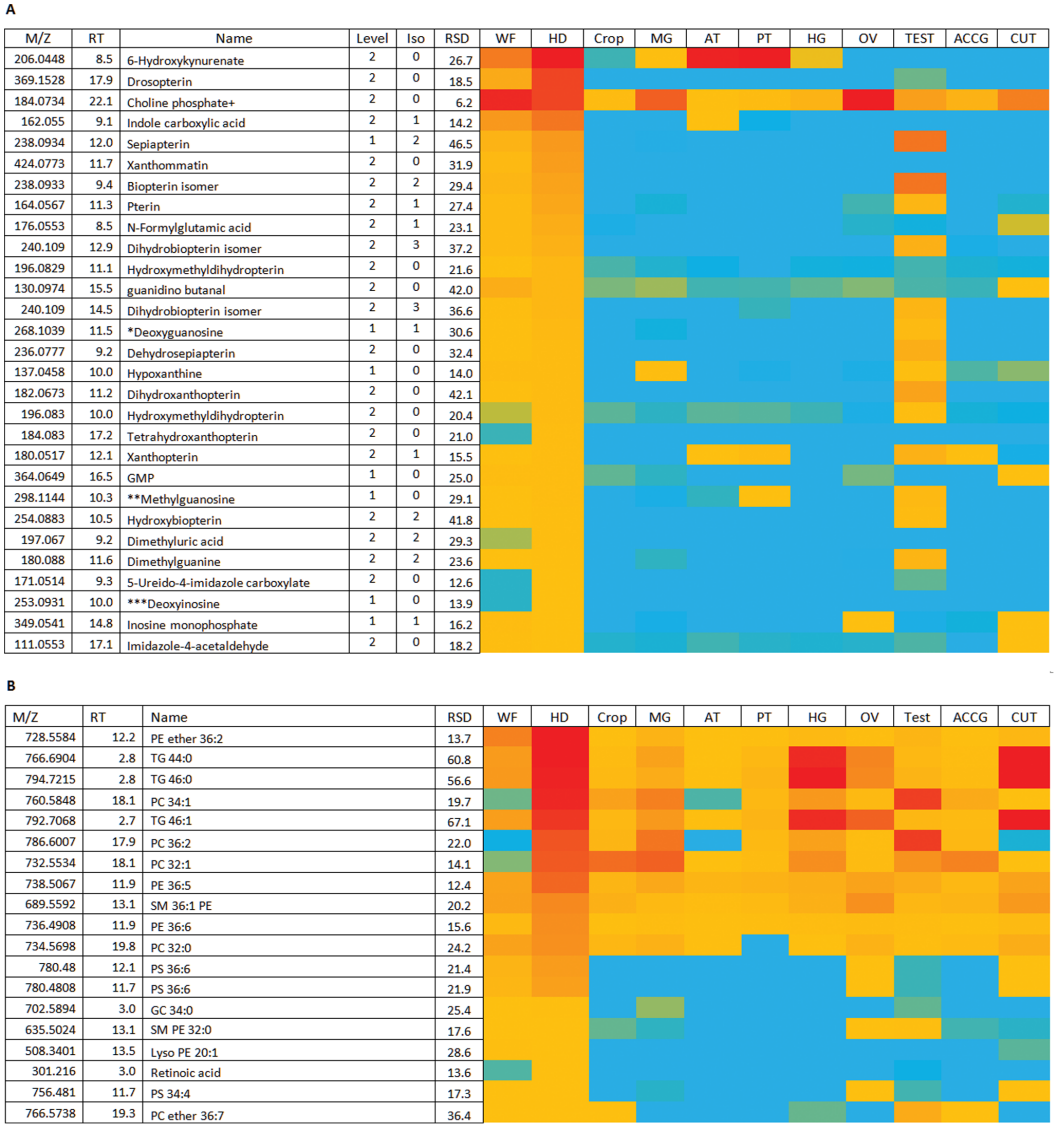
Major metabolites in *Drosophila* head. (A) Polar metabolites. *Isomeric with adenosine which but elutes earlier gives guanine as an in source fragment ion. **Gives guanine as an in source fragment ion. ***Gives inosine as a fragment. Data are presented as a heat-map of peak areas, from red (>5×10^7^) to blue (<5×10^3^). (B) Relatively abundant lipids in *Drosophila* head. PE = phosphatidylethanolamine; PC = phosphatidylcholine; PS = phosphatidyl serine; TG = Triglcyceride; SM = Sphingomyelin. Data are presented as a heat-map of peak areas, from red (>1×10^7^) to blue (<1×10^3^).

Although the tubule metabolome is depleted for most amino acids, tryptophan is a conspicuous exception (**Fig. 2**), presumably because of its role as a visual pigment precursor, and the known role of tubules in accumulating and processing pigment precursors [20]. Consistent with this finding, the allied metabolites kyurenine, hydroxykynurenine, hydroxykyurenate, formyl kynurenine and hydroxytryptophan dominate the tissue metabolome. Active transport of tryptophan into the Malpighian tubule has been previously demonstrated [21]. There is also a strong emphasis on tryptophan metabolism in the head (**Fig. 3A**). However, in the head the kynurenine pathway is used to form the terminal pigment xanthomattin [20], which is not present in the tubules. In previous work it was observed that the ratio of glutamine: glutamate was highest in the abdomen of the fly where ammonia production is greatest [6]. In **Table S3** it can be seen that it is possible to be more specific and observe that the glutamine: glutamate ratio (and thus ammonia production) is greatest in the posterior tubules, which reside in the abdomen.

### The Head Metabolome Contains High Levels of Pigments and Ether Lipids

The head sample contains the brain, but also the brightly-coloured compound eyes. As well as xanthommatin, *Drosophila* heads (Table **S3A**) show very high levels of the red pterin metabolites, consistent with the strongly head-enriched expression of pigment processing genes such as *sepia*, *Plum*, *Henna, vermillion* and *brown* (FlyAtlas.org). The pterins and biopterins are derived from GTP, and although GTP is not observed there are a number of metabolites related to guanine in the head including guanine, deoxyguanosine and GMP.

The head also displays a unique pattern of lipids, probably reflecting the unique needs of the brain (the largest single tissue in the head). One of the most abundant lipids in the head is a 36∶2 ether lipid (**Fig. 3B**). Carvalho *et al* also reported that ether lipids were abundant in *Drosophila* brain [5]; the brain composes a large part of the *Drosophila* head. Such ether lipids in the form of plasmalogens are also abundant in the human brain [22]. Carvalho *et al* also reported that the brain was abundant in triglycerides; this was also observed in the heads in our study (**Fig. 3B**).

### The Cuticle is a Site of Defence against Oxidative Stress

**Figure 4:**
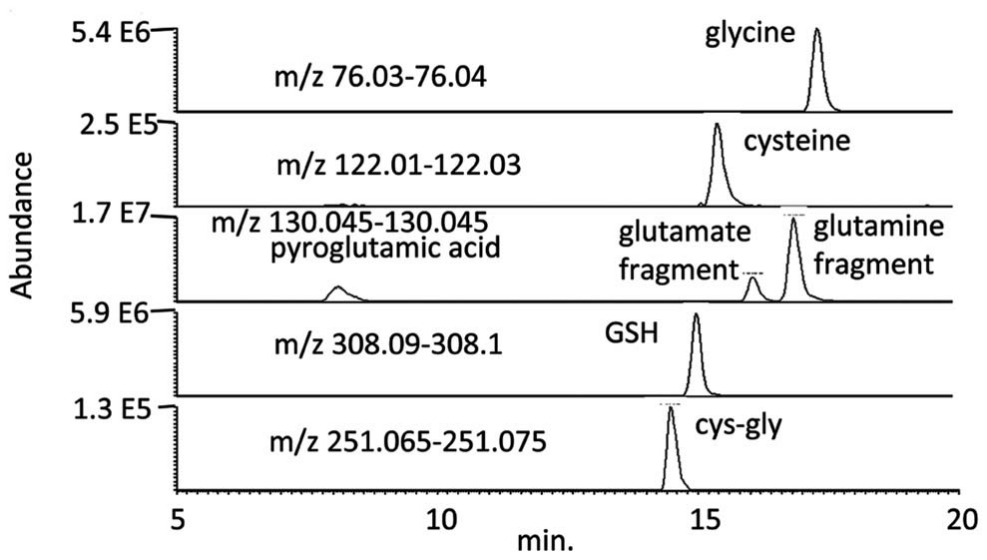
Extracted ion chromatograms showing GSH and its precursors extracted from Drosophila cuticle.

**Figure 5:**
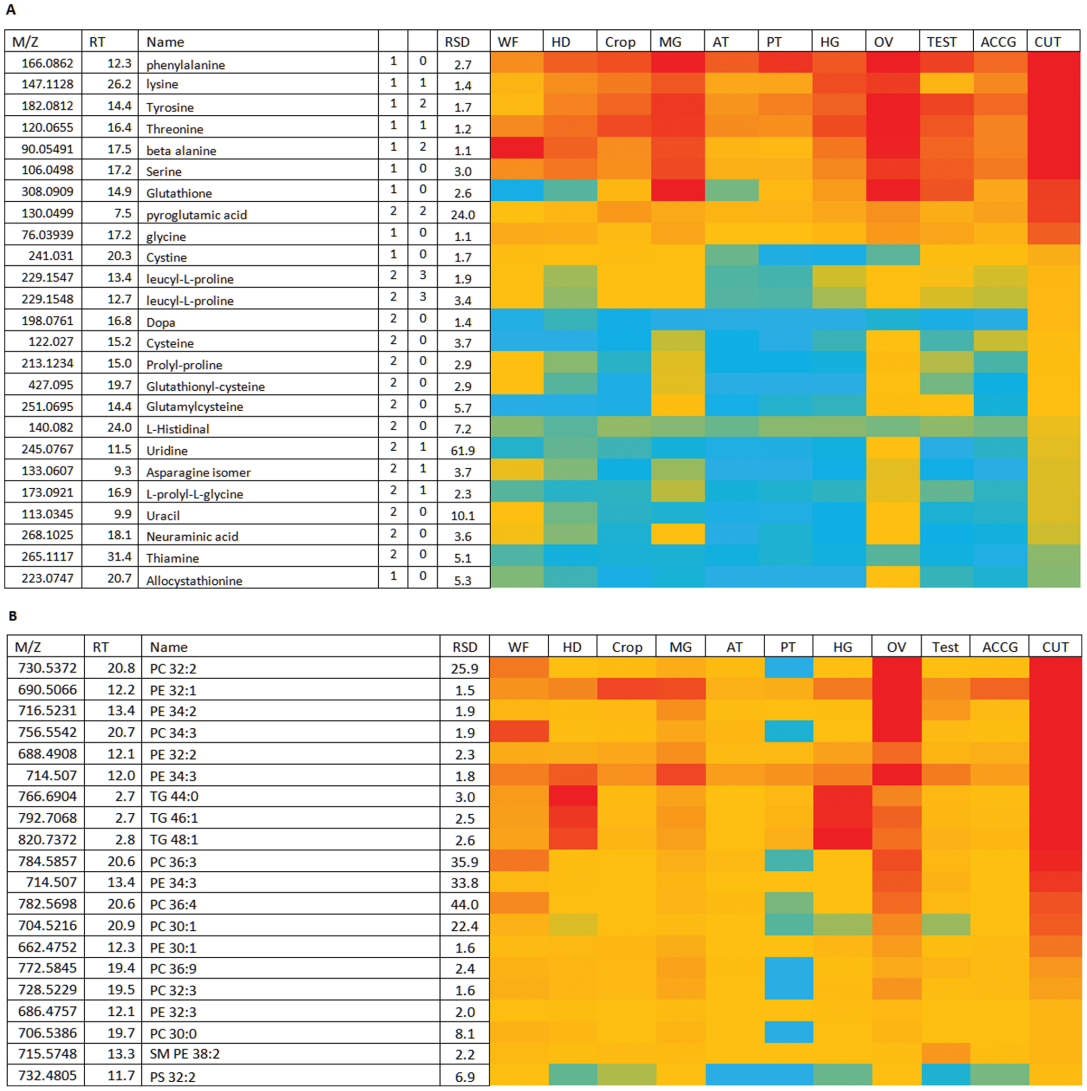
Major metabolites in cuticle. (A) Relatively abundant polar metabolites in cuticle. Data are presented as a heat-map of peak areas, from red (>2×10^7^) to blue (<5×10^3^). (B) Relatively abundant lipids in cuticle. Data are presented as a heat-map of peak areas, from red (>1×10^7^) to blue (<5×10^3^).

Insect cuticle provides a mechanical exoskeleton, and a hydrocarbon-based waxy permeability barrier against evaporative water loss. *Drosophila* cuticle has a distinctive pattern of both polar and lipid metabolites, and contains high levels of cystine and methionine S-oxide, indicative of oxidative stress. High levels of the antioxidant glutathione (GSH) are present in cuticle whereas it is barely detectable in the whole fly (**Fig. 4**). By contrast, the GSH-cysteine conjugate is abundant in whole fly, suggesting that GSH may react with some component of the whole fly during extraction (**Fig. 5A**). It would appear that GSH biosynthesis is important in the cuticle with high levels of the precursors cysteine and glycine being present in the cuticle. Glycine is a non-essential amino acid which is derived from threonine which is the most abundant metabolite in the cuticle. Glycine combines with glutamylcysteine (GC) which is formed by the combination of glutamate and cysteine to form GSH, GC can also be observed in the cuticle although its levels are higher in the other GSH rich tissues. Glycine can be a limiting precursor in GSH biosynthesis and this can cause recycling of GC back to glutamate and cysteine. Pyroglutamic acid is an intermediate in this pathway, and has been monitored in urine from neonates as an indicator of glycine deficiency [23]; pyroglutamic acid is also abundant in the cuticle **(** **Fig. 5** **)**. Glutamate is in high abundance in all tissues in *Drosophila*, however, cysteine is absent from many tissues but present in all the tissues where GSH is abundant (**Fig. 5A**).

The cuticle also contains relatively high levels of dopa and its precursor tyrosine. Dopa is important for both the production of melanin pigments and in the formation of the quinones required for the crosslinking reactions required in the formation of the insect cuticle. Tyramine which is in this pathway is also abundant in the cuticle. Insect cuticle also contains collagen proteins and in Fig. 5A it can be seen that prolylglycine and hydroxyprolyl glycine, which are produced by collagen turnover, are abundant. Fig. 5B shows the distribution of lipids in cuticle; similarly to ovary, it is particularly rich in PC and PE lipids, and triglycerides. This unusual composition, compared with the other tissues, may reflect the vital waterproofing properties of the waxy epicuticle; as insects are so small, their high surface area : volume ratio exposes terrestrial insects to severe risk of desiccation [24].

### Ovaries show Hallmarks of Lipid Biosynthesis

**Figure 6:**
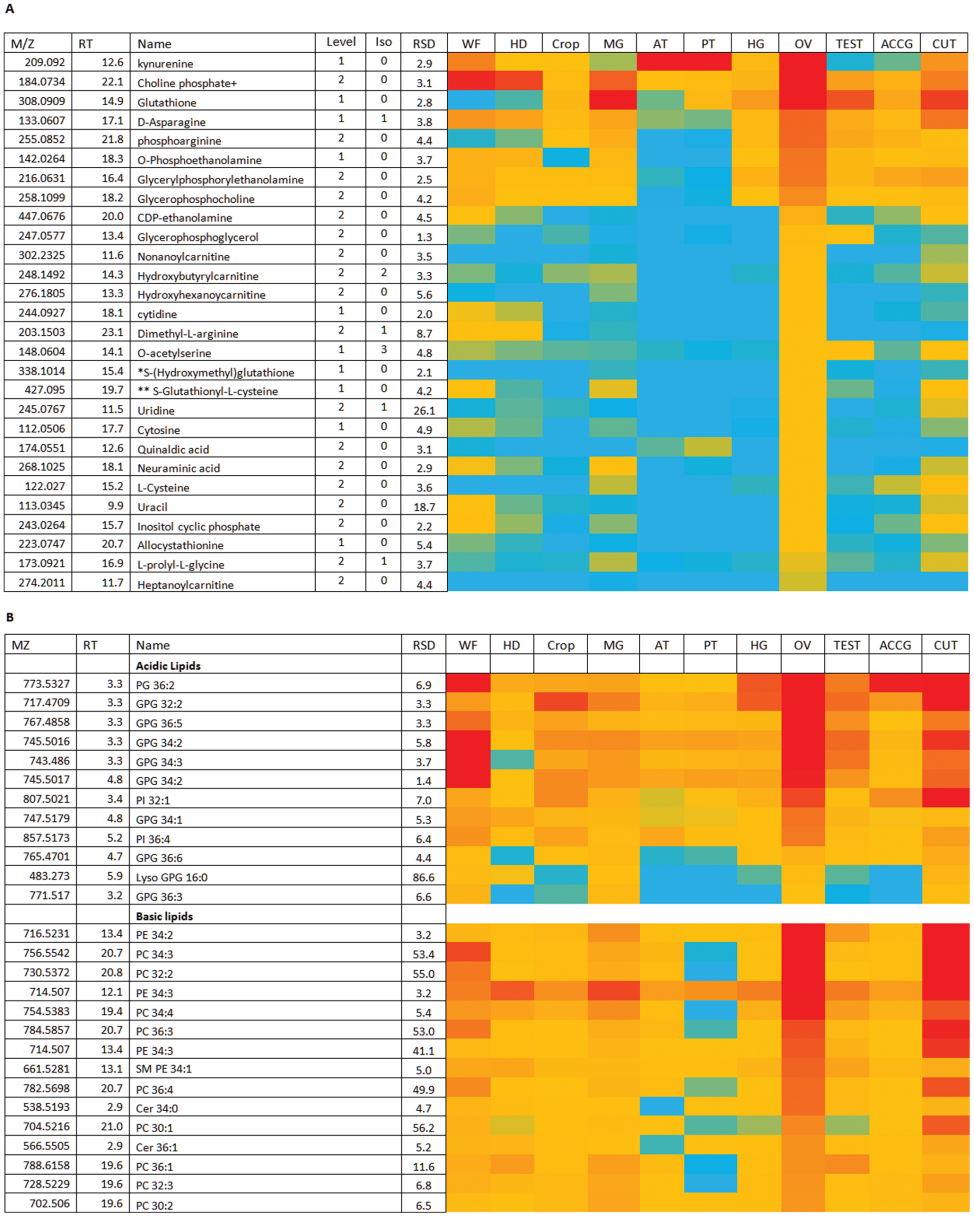
Major metabolites in ovaries. (A) Relatively abundant polar metabolites in Drosophila ovaries. *In source GSH fragment formed. **In source GSH fragment formed. Data are presented as a heat-map of peak areas, from red (>2×10^7^) to blue (<5×10^3^). (B) Relatively abundant lipids in *Drosophila* ovary. PG = phosphoglycerol; GPG = glyceryl phosphoglycerol; PI = phosphoinositol; Cer = ceramide. Data are presented as a heat-map of peak areas, from red (>1×10^7^) to blue (<1×10^3^).

The ovaries form a major fraction of the mass of the female body, and after mating (as in this sample) they are major sites of biosynthesis as many hundreds of eggs are produced over a few weeks. **Fig. 6A** shows the relatively abundant polar metabolites present in *Drosophila* ovaries. As in cuticle, the oxidative stress indicators methionine sulphoxide, hydroxymethylglutathione, GSH and glutathione-cysteine are abundant in ovary suggesting that the tissue is a site of high metabolic activity. The tissue has been reported as a highly active site for the growth and maturation of stem cells [25] and antioxidant defences are important for protecting stem cells from the effects of aging. Similarly genes involved in purine biosynthesis are abundant but there is no evidence for accumulation intermediates in purine biosynthesis in the tissues. High levels of purine and pyrimidine biosynthesis may be a reflection of the high rate of stem cell formation in this tissue.

The ovaries also contain high levels of phosphoarginine which is a high energy reserve metabolite, like creatinine phosphate, and is used to maintain ATP levels; confirming that the ovaries are very active metabolically. Phosphoarginine is normally associated with maintaining ATP levels in muscle tissue and is utilised as a high energy substrate in motile cells such as spermatozoa [26]. Cell motility is also an important component in oogenesis in *Drosophila* [27].

Kynurenine is conspicuous and hydroxykynurenine is also relatively abundant. The kynurenine pathway is involved in egg pigmentation in the silk moth *Bombyx mori* [28] but in *Drosophila* ovaries there is no indication of the downstream formation of xanthomattin pigments. Kynurenine may thus be accumulating as a local end-product of ovarian tryptophan metabolism.

The ovaries are abundant in metabolites such as phosphoethanolamine, glycerolphosphoethanolamine, CDP-ethanolamine, choline phosphate and the polar precursor glycerophosphoglycerol (GPG) suggesting that lipid biosynthesis is very active in this tissue (**Fig. 6B**). The nurse cells in Drosophila ovaries are known to store lipid droplets [29]. However, in flies genetically deficient in germ cells, little difference in overall lipid profile is observed [30].

The other pathway that is highly expressed in ovary is terpenoid biosynthesis. *Drosophila* are auxotrophs for the main steroid precursor cholesterol and thus it is unlikely flux through this pathway is due to steroid biosynthesis.

### Testes Contain High Levels of Pterins and Ether Lipids

**Figure 7:**
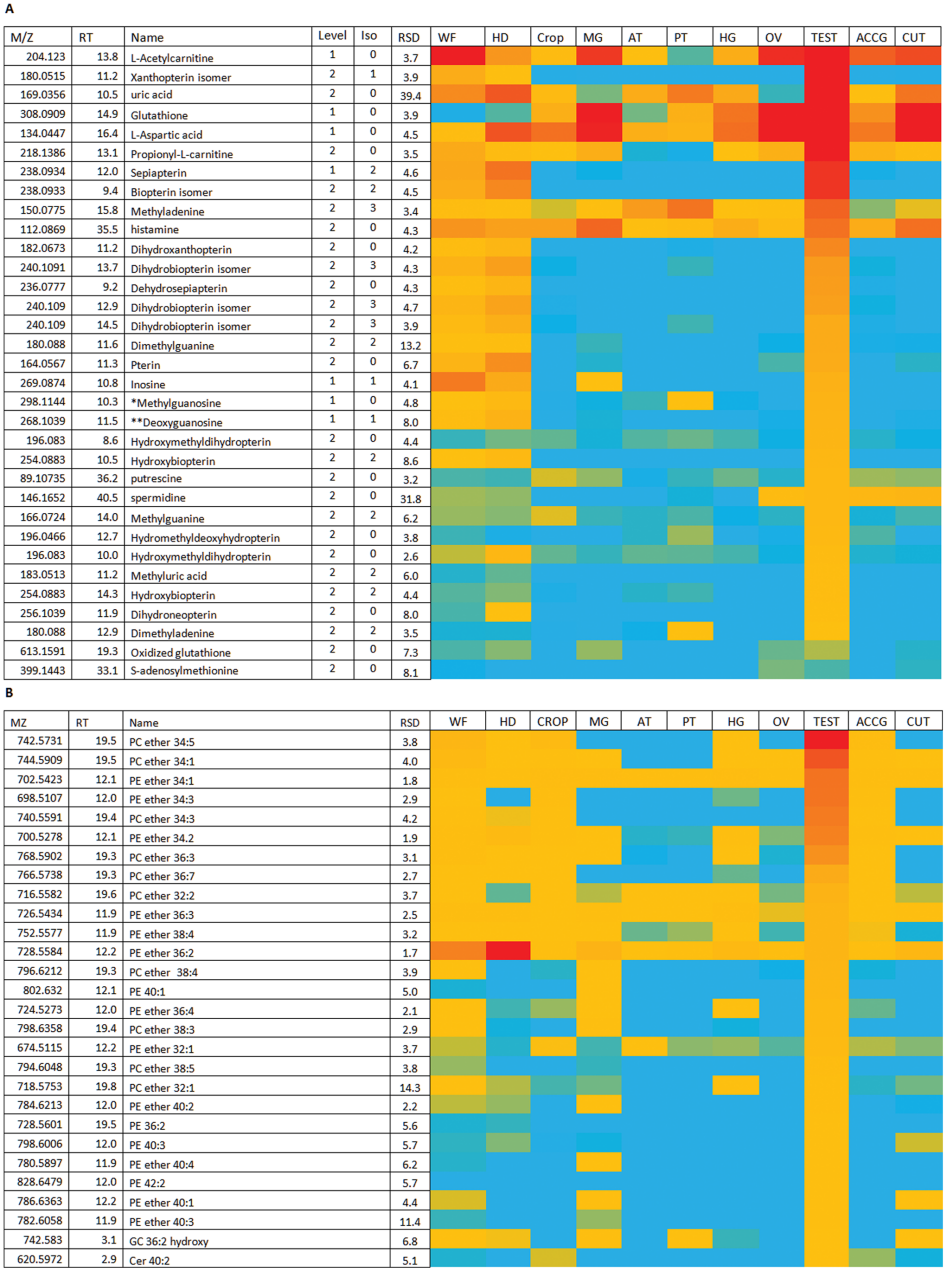
Major metabolites in testes. (A) Testes relatively abundant polar metabolites. *Gives guanine as an in source fragment ion. **Isomeric with adenosine which but elutes earlier gives guanine as an in source fragment ion. Data are presented as a heat-map of peak areas, from red (>1×10^7^) to blue (<5×10^3^). (B) Relatively abundant lipids in testes. Data are presented as a heat-map of peak areas, from red (>1×10^7^) to blue (<1×10^3^).

The testis transcriptome is the most unique and divergent in the fly, reflecting the unique demands of producing huge numbers of semiautonomous haploid, motile sperm [31]. Testes contain similar levels of eye pigment-associated pterin, xanthopterin and biopterins to those found in *Drosophila* heads (**Fig. 7A**). Although the presence of pterins in the testis sheath has been documented [32], their role in the testes is unknown. Testes are also one of the tissues with high levels of GSH suggesting protection against oxidative stress is important in this tissue. Phosphoarginine levels are also high in testes, reflecting its requirement for the motility of spermatozoa [26]. The lipid pattern in testes is very distinctive (**Fig. 7B**), with high levels of PE, PC and particularly ether lipids, major components in the membranes of spermatozoa [33]. The most abundant polar metabolite in testes is carnitine closely followed by acetyl carnitine and these two metabolites are known to be abundant in spermatozoa and to possibly have a role in sperm motility [34].

### The Midgut is Rich in Carnitines

**Figure 8:**
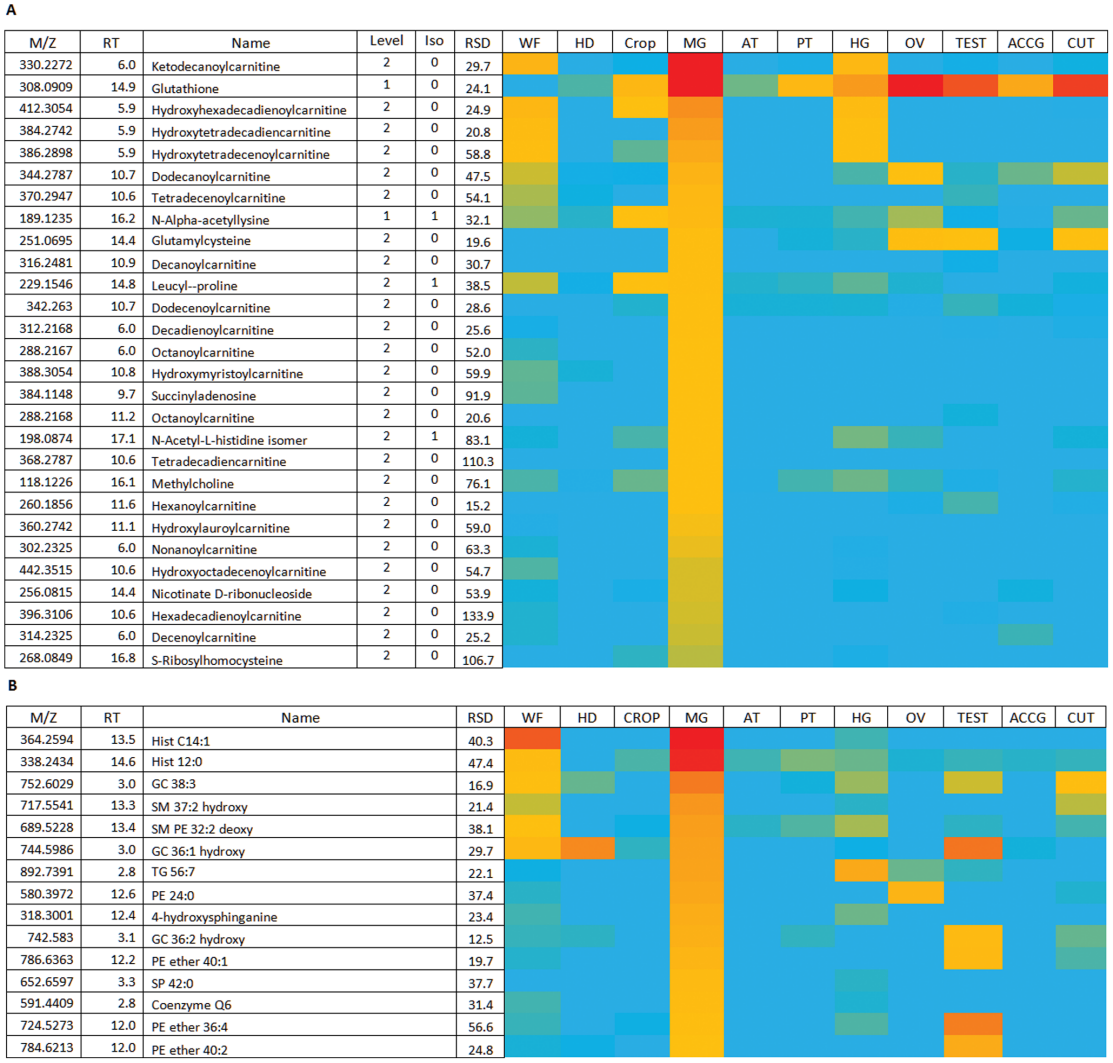
Major metabolites in midgut. (A) Relatively abundant metabolites in midgut. Data are presented as a heat-map of peak areas, from red (>1×10^7^) to blue (<5×10^3^). (B) Relatively abundant lipids in the midgut. Hist = acyl histamine. Red = area >10^7^ Yellow area >2×10^4^ Blue <10^3^.

The midgut is a permeable, metabolically active tissue, in which most digestion and nearly all nutrient absorption takes place [35]. **Fig. 8A** shows the relatively abundant metabolites in midgut which is another tissue which is high in GSH. The high GSH levels in the mid-gut probably result from it being a site of oxidative stress and the integrity of the midgut barrier in *Drosophila* has been associated with longevity [36], [37].

Most uniquely, the midgut also contains high levels of a range of acylcarnitines, which are involved in moving fatty acids in and out of the mitochondria and act to buffer the levels of free CoA within the mitochondria. These compounds are absent from the other tissues. This ties in nicely with the known transcriptome of the midgut [16]; the midgut is the unique site of expression of most dietary lipases, and so could be expected to be the major site of uptake and processing of fatty acids. Enzymes involved in carnitine biosynthesis are abundant in gut tissue in mice [38]; however, carnitine is an essential nutrient for insects, and indeed was originally characterized as a novel vitamin (B_T_) in mealworms [39].

The lipid composition of midgut is generally similar to many of the other tissues, but as reported previously in larvae [5] sphingolipids, and here sphinganine, phytosphinganine, sphingomyelins, ceramide and glycosylceramides are relatively abundant (**Fig. 8B**). The sphingolipids serve a barrier function; particularly hydroxylated sphingolipids which pack densely [5]. These specialized lipids may help to maintain an impermeable barrier that is resistant to the digestive lipases so abundant in midgut; indeed, the hind-gut (HG) similary shows up-regulation of genes involved in sphingolipid metabolism although the metabolites in this class are upregulated to a lesser extent than in mid-gut. Like the midgut, in hindgut, genes involved in oxidative stress are also up-regulated, and HG contains relatively high levels of GSH. HG also shows abundant acyl carnitines. The both these tissues of the alimentary canal show, despite their distinct origins (ectodermal for the hindgut, endodermal for the midgut), common metabolic fingerprints associated with fatty acid metabolism, oxidative stress response, and barrier functions.

### N-acetylhistamine and Methionine in Accessory Gland

**Figure 9:**
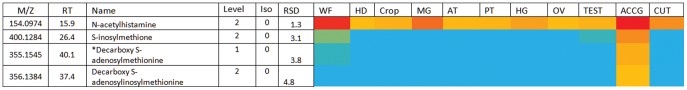
Relatively abundant polar metabolites in male accessory glands. *In source fragment for methylthioadenosine. Data are presented as a heat-map of peak areas, from red (>1×10^7^) to blue (<1×10^3^).

The accessory gland provides specialized proteinaceous secretate to accompany the sperm on their voyage, in order to modulate the fertility of the female fly to increase egg-laying and prevent fertilisation by another male [40]. Perhaps surprisingly, we found relatively few metabolic sequelae of this unique function. The lipid profile (not shown) was unremarkable, and **Fig. 9** shows that relatively few polar metabolites were conspicuous in accessory gland (ACCG). N-acetylhistamine is highly abundant in this tissue compared to other tissues and it is conceivable, since histamine has been proposed as a neurotransmitter in *Drosophila* [41], that hydrolysis of N-acetylhistamine to histamine might have marked effects. As the only known reaction to feature N-acetyl histamine is the deacetylation of acetyl CoA by histidine, n-acetylhistamine may simply be accumulating as an end-product of metabolism unique to the accessory gland.

**Figure 10:**
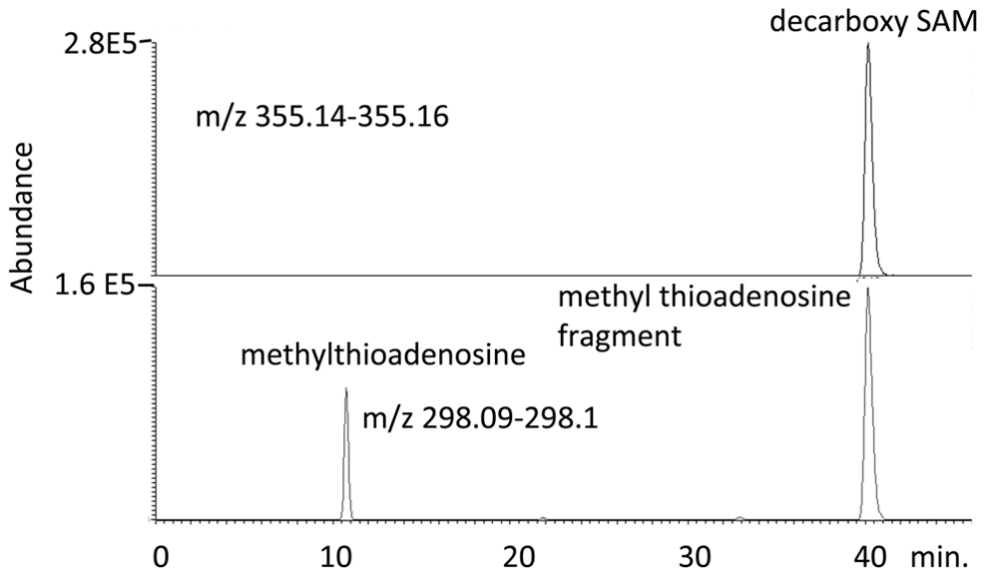
Extracted ion traces for decarboxy S-adenosylmethione and its fragment methylthioadenosine, a metabolite unique to accessory glands.

The accessory gland also uniquely shows high levels of certain methionine derivatives, notably decarboxylated S-adenosylmethionine (**Fig. 9**). This molecule is quite characteristic since it forms an in source fragment corresponding to methylthioadenosine (**Fig. 10**). In addition as would be predicted the absence of a carboxyl group in the molecule gives it a later retention time than SAM [42]. In fact the picture is further complicated by the presence of two compounds which appear to be the inosine analogues of S-adenosylmethionine and decarboxy S-adenosylmethionine. S-adenosylmethionine is not detectable in ACCG tissue but can be observed in low levels in some other tissues such as testes. The function of these compounds is unknown although it would seem likely that they would be involved in polyamine biosynthesis.

### Crop

**Figure 11:**
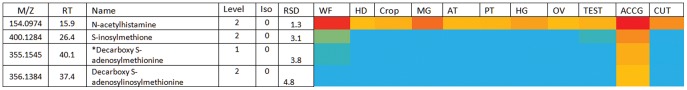
Relatively abundant lipids in crop. PA = phosphatidic acid. Data are presented as a heat-map of peak areas, from red (>1×10^7^) to blue (<5×10^3^).

The crop is a diverticulum of the foregut, which is classically considered to be a highly impermeable, cuticle-lined storage organ [43]. In flies, it is used to store nectar meals, so that it can be assimilated gradually. There is nothing particularly distinctive about the pattern of polar metabolites in the crop tissue (not shown). However, as can be seen in **Fig. 11** the crop is moderately abundant in PI lipids. PI lipids have an important role in cell movement [44] and the abundance of these lipids in the crop may related to the presence of stretch receptors in this tissue which are important for regulating food intake [45].

### Lysolipids in the Whole Fly

**Figure 12:**
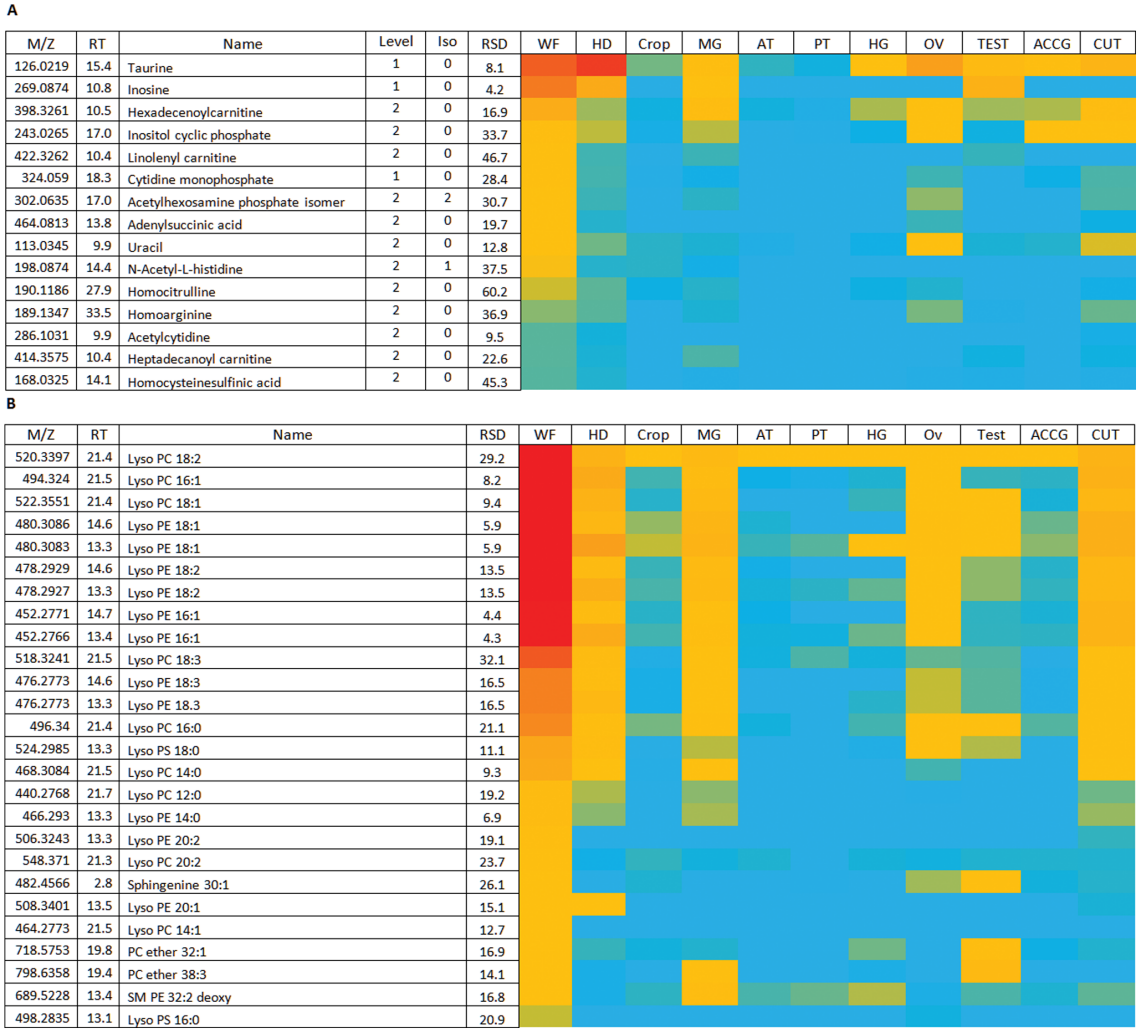
Major metabolites of whole fly. (A) Relatively abundant polar metabolites in whole fly. Data are presented as a heat-map of peak areas, from red (>1×10^7^) to blue (<1×10^3^). (B). The predominance of lysolipids in whole fly. Data are presented as a heat-map of peak areas, from red (>1×10^7^) to blue (<1×10^3^).

As it represents a mass-weighted average of the major tissues, there are only a few distinguishing metabolites in the whole fly that have not already been discussed (**Fig. 12**). Homoarginine and homocitrulline are much higher in whole fly than any of the tissues. By contrast, the lipidome shows a unique profile, with higher levels of a range of lysolipids than seen in any individual tissue. This probably reflects abundant expression in some tissue that we have yet to dissect and profile; in the analogous FlyAtlas.org expression resource [16], such whole-animal outliers (higher whole animal expression than would be predicted from the ca. 5% mRNA contributions of the individual tissues), vanished as further tissues were profiled. There is no reason to think that this will not be the case for metabolomes.

## Conclusion

These results are useful at several levels. Firstly, the non-uniform enrichments of particular metabolites across different tissues, provide validation of the methodology (where they correspond to already known functions of the tissues), or useful hypothesis-free insights for further experimental investigation. Critically for the field, these data not only provide the first atlas of metabolites across multiple tissues in this important model, but also shows that the metabolomes of each tissue differ markedly and repeatably, both from each other, and from the ‘global average’ of the whole organism metabolome. As with transcriptomics [16], it is thus not sufficient to grind up the whole organism to perform metabolomic analysis! The usefulness of the resource will also increase further as parallel analysis techniques increase the number of classes of measurable metabolites. In the longer term, this resource can provide a baseline against which to assess experimental manipulations – for example, dietary alterations in the study of aging or the role of mutations in tissue and organism function.

## Supporting Information

Table S1 Protein normalisation factors for each tissue.
(DOCX)

Table S2 Retention time of standard compounds.
(DOCX)

Table S3 Two hundred and forty two putatively identified polar metabolites ranked according to their abundance in whole fly. All metabolites were within ±1.5 ppm of the exact mass of the metabolite in the database.
(DOCX)

Table S4 Positively charged lipids in Drosophila ranked according to abundance in the whole fly.
(DOCX)

Table S5 Putatively identified negatively charged lipids in Drosophila tissues ranked according to abundance in the whole fly. Mass deviation from the exact masses in the database were <2 ppm.
(DOCX)
