# Supplementary material for: Mapping an Atlas of Tissue-Specific Drosophila melanogaster Metabolomes by High Resolution Mass Spectrometry
Source: PLoS One. 2013 Oct 29;8(10):e78066. doi: 10.1371/journal.pone.0078066 (PMC3812166; doi:10.1371/journal.pone.0078066)
Supplement: Table S1 — Protein normalisation factors for each tissue. (DOCX) [file pone.0078066.s001.docx]

**Supplementary Table 1: Protein normalisation factors for each tissue.**

| **tissues** | **Protein content (*μ*g)** | Normalization factor |
| --- | --- | --- |
| Whole fly | 14.886216 |  |
| Whole fly | 14.975676 |  |
| Whole fly | 14.886216 | 1.00 |
| Head | 4.013456 |  |
| Head | 4.639738 |  |
| Head | 4.729208 | 3.36 |
| Ovary | 2.893964 |  |
| Ovary | 2.811949 |  |
| Ovary | 2.811949 | 5.24 |
| Accessory gland (M) | 2.029094 |  |
| Accessory gland (M) | 2.058918 |  |
| Accessory gland (M) | 1.947081 | 7.40 |
| Testis | 2.111108 |  |
| Testis | 2.126019 |  |
| Testis | 2.282591 | 6.86 |
| Cuticle | 2.021639 |  |
| Cuticle | 1.909801 |  |
| Cuticle | 2.044006 | 7.48 |
| Anterior tubules | 1.768143 |  |
| Anterior tubules | 1.522103 |  |
| Anterior tubules | 1.768143 | 8.87 |
| Posterior tubules | 1.440089 |  |
| Posterior tubules | 1.171683 |  |
| Posterior tubules | 1.440089 | 10.00 |
| Hindgut | 1.514646 |  |
| Hindgut | 1.410266 |  |
| Hindgut | 1.768143 | 9.60 |
| Crop | 1.529558 |  |
| Crop | 1.492279 |  |
| Crop | 1.410266 | 10.09 |
| Midgut | 2.327324 |  |
| Midgut | 2.327324 |  |
| Midgut | 2.357148 | 6.37 |

Three separate protein determinations for each tissue are shown, together with an average normalization factor, based on protein content of the whole fly.
