# Supplementary material for: Mapping an Atlas of Tissue-Specific Drosophila melanogaster Metabolomes by High Resolution Mass Spectrometry
Source: PLoS One. 2013 Oct 29;8(10):e78066. doi: 10.1371/journal.pone.0078066 (PMC3812166; doi:10.1371/journal.pone.0078066)
Supplement: Table S2 — Retention time of standard compounds. (DOCX) [file pone.0078066.s002.docx]

**Supplementary table 2**

Retention time of standard compounds

| **Compound Name** | **Formula** | **Polarity** | **Detected m/z** | **Delta (ppm)** | **RT** |
| --- | --- | --- | --- | --- | --- |
| **N-acetylaspartate** | C6H10NO5 | + | 170.0553 | -0.14 | 7.9 |
| **O-Acetylcarnitine** | C9H17NO4 | + | 204.1231 | 0.43 | 13.6 |
| **N-Acetyl-D-Glucosamine** | C8H15NO6 | + | 222.0972 | -0.27 | 11.8 |
| **N-Acetyl-D-glucosamine 6-phosphate** | C8H16NO9P | + | 302.0635 | -0.32 | 14.6 |
| **N6-Acetyl-L-Lysine** | C8H16N2O3 | + | 189.1233 | -0.25 | 16.6 |
| **N-Acetyl-D-mannosamine** | C8H15NO6 | - | 220.0832 | 2.34 | 12.0 |
| **O-Acetylserine** | C5H9NO4 | + | 14.0606 | 0.78 | 13.6 |
| **Adenine** | C5H5N5 | + | 136.0618 | 0.31 | 14.4 |
| **Adenosine** | C10H13N5O4 | + | 268.104 | -0.1 | 12.9 |
| **Adrenaline** | C9H13NO3 | + | 184.0968 | -0.02 | 16.7 |
| **Alanine** | C3H7NO2 | + | 90.05499 | 0.42 | 16.3 |
| **Allantoin** | C4H6N4O3 | - | 157.0369 | 1.26 | 12.8 |
| **3-aminobutyrate** | C4H10NO3 | + | 104.0706 | -0.41 | 16.3 |
| **Aminolevulinate** | C5H9NO3 | + | 132.0654 | -0.8 | 17.7 |
| **AMP** | C10H14N5O7P | + | 348.0703 | -0.31 | 16.1 |
| **dAMP** | C10H14N5O6P | - | 330.0616 | 2.1 | 15.2 |
| **L-Arabinose** | C5H10O5 | - | 149.0457 | 1.23 | 12.34 |
| **Arginine** | C6H14N4O2 | + | 175.119 | 0.12 | 25.9 |
| **Asparagine** | C4H9N2O3 | + | 133.0607 | -0.37 | 17.2 |
| **Aspartate** | C4H7NO4 | + | 134.0445 | -2.39 | 16.5 |
| **Beta alanine** | C3H7NO2 | + | 90.05499 | 0.39 | 17.4 |
| **Betaine** | C5H11NO2 | + | 118.0862 | -0.75 | 14.8 |
| **Biopterin** | C9H11N5O3 | + | 238.0934 | -0.34 | 7.8 |
| **Biotin** | C10H16N2O3S | + | 245.0954 | -0.36 | 7.9 |
| **Carnitine** | C7H16NO3 | + | 162.1125 | -0.06 | 16.2 |
| **Cis-Aconitate** | C6H6O6 | - | 173.0095 | 1.82 | 8.0 |
| **Citrate** | C6H8O7 | - | 191.02 | 1.22 | 11.3 |
| **CMP** | C9H14N3O8P | - | 323.045 | 1.17 | 17.9 |
| **Creatinine** | C4H7N3O | + | 114.0662 | -0.23 | 15.6 |
| **Cytosine** | C4H5N3O | + | 112.0505 | -0.01 | 17.2 |
| **Cystathionine** | C7H14N2O4S | + | 223.0745 | -1.08 | 20.7 |
| **Cystine** | C6H12N2O4S2 | + | 241.0307 | -1.82 | 20.4 |
| **Cytidine** | C9H13N3O5 | + | 244.0842 | -35.17 | 17.8 |
| **Dopamine** | C8H11NO2 | + | 154.0862 | -0.08 | 14.9 |
| **Ethanolamine phosphate** | C2H8NO4P | - | 140.012 | 0.93 | 19.2 |
| **Fructose** | C6H12O6 | - | 179.0564 | 1.42 | 12.7 |
| **Fucose** | C6H12O5 | - | 163.0615 | 1.54 | 11.1 |
| **Fumarate** | C4H4O4 | - | 115.0037 | 0.43 | 7.3 |
| **Galactose** | C6H12O6 | - | 179.0563 | 0.91 | 14.4 |
| **Glucosamine** | C16H14NO5 | + | 180.0866 | 0.06 | 21.0, 21.6 |
| **Glucosamine 6-Phosphate** | C6H14NO8P | + | 260.0527 | -0.94 | 21.5 |
| **Glucose** | C6H12O6 | - | 179.0563 | 1.17 | 14.0 |
| **Glucuronate** | C6H10O7 | - | 193.0354 | 0.13 | 16.5 |
| **Glutamate** | C5H9NO4 | + | 148.0604 | -0.27 | 16.0 |
| **Glutamine** | C5H11N2O3 | + | 147.0764 | -0.22 | 16.7 |
| **Glutathione** | C10H17N3O6S | - | 306.0772 | 2.22 | 14.8 |
| **Glycine** | C2H5NO2 | + | 76.03934 | 0.52 | 17.3 |
| **GMP** | C10H14N5O8P | + | 364.0651 | -0.41 | 15.8 |
| **Guanine** | C5H5N5O | + | 152.0567 | 0.1 | 14.0 |
| **Guanosine** | C10H13N5O5 | + | 284.0989 | -0.35 | 12.5 |
| **Histidine** | C6H9N3O2 | + | 156.0768 | 0.48 | 24.0 |
| **Homocysteine** | C4H9NO2S | + | 136.0425 | -1.1 | 20.4 |
| **Homogentisate** | C8H8O4 | - | 167.0351 | 0.77 | 7.8 |
| **Homoserine lactone** | C4H7NO2 | + | 102.055 | 0.59 | 17.2 |
| **5-Hydroxyindoleacetate** | C10H9NO3 | + | 192.0654 | -0.55 | 7.9 |
| **Hydroxyproline** | C5H9NO3 | + | 132.0653 | -1.95 | 17.0 |
| **Hypoxanthine** | C5H4N4O | + | 137.0459 | 0.7 | 9.9 |
| **Imidazole-4-acetate** | C5H6N2O2 | + | 127.0501 | -1.1 | 14.9 |
| **IMP** | C10H13N4O8P | - | 347.0404 | 1.68 | 15.9 |
| **Inosine** | C10H12N4O5 | + | 269.088 | -0.24 | 10.7 |
| **Isocitrate** | C6H8O7 | - | 191.0201 | 1.78 | 12.2 |
| **Isonicotinic acid** | C6H5NO2 | + | 124.0393 | -0.17 | 9.4 |
| **Isoleucine** | C6H13NO2 | + | 132.1019 | -0.41 | 12.8 |
| **Kynurenine** | C10H12N2O3 | + | 209.0916 | -2.1 | 10.5 |
| **Leucine** | C6H13NO2 | + | 132.1020 | 0.11 | 12.5 |
| **Lysine** | C6H14N2O2 | + | 147.1128 | -0.37 | 26.4 |
| **Malonate** | C3H4O4 | - | 103.0037 | -0.19 | 15.9 |
| **Maleic acid** | C4H4O4 | - | 115.0037 | 0.29 | 13.2 |
| **Maltose** | C12H22O11 | - | 341.1098 | 2.63 | 15.2 |
| **Mannose** | C6H12O6 | - | 179.0565 | 2.02 | 13.1 |
| **Metanephrine** | C10H15NO3 | + | 198.1121 | -2.14 | 17.2 |
| **Methionine** | C5H12NO2S | + | 150.0584 | 0.63 | 13.2 |
| **N(pi)-Methyl-L-histidine** | C7H11N3O2 | + | 170.0965 | 0.22 | 24.6 |
| **Methylmalonate** | C4H6O4 | - | 117.0192 | -0.77 | 14.9 |
| **5'-Methylthioadenosine** | C11H15N5O3S | + | 298.0967 | -0.53 | 10.7 |
| **Nicotinamide** | C6H7N2O | + | 123.0552 | -0.48 | 9.4 |
| **NAD+** | C21H27N7O14P2 | + | 664.1168 | 0.64 | 20.5 |
| **Nicotinate** | C6H5NO2 | + | 124.0393 | -0.11 | 8.3 |
| **Ornithine** | C5H13N2O2 | + | 133.0971 | -0.40 | 26.4 |
| **2-Oxoglutarate** | C5H6O5 | - | 145.0143 | 0.67 | 11.0 |
| **5-Oxoproline** | C5H7NO3 | + | 130.0498 | -0.38 | 8.1 |
| **Pantothenate** | C9H17NO5 | + | 220.1178 | -0.62 | 7.9 |
| **Phenylalanine** | C9H11NO2 | + | 166.0862 | -0.08 | 12.1 |
| **Phosphoenolpyruvate** | C3H5O6P | - | 166.9752 | 0.73 | 17.0 |
| **Picolinic acid** | C6H5NO2 | + | 124.0392 | -0.79 | 6.2 |
| **Proline** | C5H10NO2 | + | 116.0706 | 0.042 | 14.6 |
| **Riboflavin** | C17H21N4O6 | + | 377.1457 | 0.30 | 8.1 |
| **Sarcosine** | C3H7NO2 | + | 90.05494 | -0.18 | 16.5 |
| **Sepiapterin** | C9H11N5O3 | + | 238.0934 | -0.34 | 11.6 |
| **Serine** | C3H8NO3 | + | 106.0499 | 0.46 | 17.0 |
| **Sorbitol** | C6H14O6 | + | 183.0862 | -0.64 | 14.8 |
| **Succinate** | C4H6O4 | - | 117.0195 | 1.51 | 7.9 |
| **Sucrose** | C12H22O11 | + | 343.1234 | -0.22 | 13.1 |
| **Taurine** | C2H8NSO3 | + | 122.0230 | 0.23 | 15.3 |
| **Threonine** | C4H10NO3 | + | 120.0655 | 0.086 | 16.4 |
| **Thymidine** | C10H14N2O5 | + | 243.0974 | -0.56 | 7.9 |
| **Tryptophan** | C11H12N2O2 | + | 205.0899 | 0.71 | 12.5 |
| **Tyrosine** | C9H11NO3 | + | 182.0815 | 1.8 | 14.2 |
| **Valine** | C5H11NO2 | + | 118.0862 | -0.55 | 14.0 |
| **UMP** | C9H14N2O9P | + | 325.0432 | 0.11 | 14.4 |
| **UDP-N-acetyl-D-glucosamine** | C17H27N3O17P2 | + | 608.0892 | 0.54 | 19.9 |
| **Xanthine** | C5H4N4O2 | - | 151.0262 | 0.03 | 10.5 |
| **D-Xylose** | C5H10O5 | - | 149.0457 | 1.03 | 12.0 |
