# Supplementary material for: Mapping an Atlas of Tissue-Specific Drosophila melanogaster Metabolomes by High Resolution Mass Spectrometry
Source: PLoS One. 2013 Oct 29;8(10):e78066. doi: 10.1371/journal.pone.0078066 (PMC3812166; doi:10.1371/journal.pone.0078066)
Supplement: Table S3 — Two hundred and forty two putatively identified polar metabolites ranked according to their abundance in whole fly. All metabolites were within ±1.5 ppm of the exact mass of the metabolite in the database. (DOCX) [file pone.0078066.s003.docx]

**Table S3** Two hundred and forty two putatively identified polar metabolites ranked according to their abundance in whole fly. All metabolites were within ±1.5 ppm of the exact mass of the metabolite in the database.

| M/Z | RT | Name | WF | HD | Crop | MG | AT | PT | HG | OV | TEST | ACCG | CUT |
| --- | --- | --- | --- | --- | --- | --- | --- | --- | --- | --- | --- | --- | --- |
| 116.0705 | 15.1 | D-Proline |  |  |  |  |  |  |  |  |  |  |  |
| 156.0767 | 24.0 | L-Histidine |  |  |  |  |  |  |  |  |  |  |  |
| 90.05495 | 16.3 | Alanine |  |  |  |  |  |  |  |  |  |  |  |
| 147.0763 | 16.8 | glutamine |  |  |  |  |  |  |  |  |  |  |  |
| 90.05491 | 17.5 | beta alanine |  |  |  |  |  |  |  |  |  |  |  |
| 175.119 | 25.8 | L-Arginine |  |  |  |  |  |  |  |  |  |  |  |
| 148.0603 | 16.0 | glutamate |  |  |  |  |  |  |  |  |  |  |  |
| 166.0532 | 16.6 | Methionine sulfoxide |  |  |  |  |  |  |  |  |  |  |  |
| 118.0862 | 15.3 | betaine |  |  |  |  |  |  |  |  |  |  |  |
| 184.0734 | 22.1 | Choline phosphate+ |  |  |  |  |  |  |  |  |  |  |  |
| 162.1124 | 16.5 | (S)-Carnitine |  |  |  |  |  |  |  |  |  |  |  |
| 132.1018 | 12.9 | D-Leucine/isoleucine |  |  |  |  |  |  |  |  |  |  |  |
| 225.0869 | 14.0 | 3-Hydroxy-L-kynurenine |  |  |  |  |  |  |  |  |  |  |  |
| 118.0862 | 14.4 | valine |  |  |  |  |  |  |  |  |  |  |  |
| 104.107 | 17.4 | Choline |  |  |  |  |  |  |  |  |  |  |  |
| 204.123 | 13.8 | L-Acetylcarnitine |  |  |  |  |  |  |  |  |  |  |  |
| 154.0974 | 15.9 | N-acetylhistamine |  |  |  |  |  |  |  |  |  |  |  |
| 206.0448 | 8.5 | 6-Hydroxykynurenate |  |  |  |  |  |  |  |  |  |  |  |
| 209.092 | 12.6 | kynurenine |  |  |  |  |  |  |  |  |  |  |  |
| 120.0655 | 16.4 | D-Threonine |  |  |  |  |  |  |  |  |  |  |  |
| 106.0498 | 17.2 | D-Serine |  |  |  |  |  |  |  |  |  |  |  |
| 166.0862 | 12.3 | phenylalanine |  |  |  |  |  |  |  |  |  |  |  |
| 126.0219 | 15.4 | Taurine |  |  |  |  |  |  |  |  |  |  |  |
| 348.0703 | 16.5 | Adenosine 2'-phosphate |  |  |  |  |  |  |  |  |  |  |  |
| 104.0706 | 16.8 | aminobutyric acid |  |  |  |  |  |  |  |  |  |  |  |
| 133.0607 | 17.1 | D-Asparagine |  |  |  |  |  |  |  |  |  |  |  |
| 162.055 | 9.1 | Indole carboxylic acid (Isomer 4,6-Dihydroxyquinoline) |  |  |  |  |  |  |  |  |  |  |  |
| 269.0874 | 10.8 | Inosine |  |  |  |  |  |  |  |  |  |  |  |
| 150.0583 | 13.5 | Methionine |  |  |  |  |  |  |  |  |  |  |  |
| 104.0707 | 16.0 | aminobutyric acid |  |  |  |  |  |  |  |  |  |  |  |
| 169.0356 | 10.5 | uric acid |  |  |  |  |  |  |  |  |  |  |  |
| 173.021 | 15.1 | Glycerol 3-phosphate |  |  |  |  |  |  |  |  |  |  |  |
| 369.1528 | 17.9 | Drosopterin |  |  |  |  |  |  |  |  |  |  |  |
| 112.0869 | 35.5 | histamine |  |  |  |  |  |  |  |  |  |  |  |
| 76.03939 | 17.2 | glycine |  |  |  |  |  |  |  |  |  |  |  |
| 205.0972 | 13.0 | D-Tryptophan |  |  |  |  |  |  |  |  |  |  |  |
| 130.0974 | 15.5 | guanidino butanal |  |  |  |  |  |  |  |  |  |  |  |
| 258.1099 | 18.2 | Glycerophosphocholine |  |  |  |  |  |  |  |  |  |  |  |
| 216.0631 | 16.4 | Glycerylphosphorylethanolamine |  |  |  |  |  |  |  |  |  |  |  |
| 170.0925 | 24.2 | 1-Methylhistidine |  |  |  |  |  |  |  |  |  |  |  |
| 142.0264 | 18.3 | O-Phosphoethanolamine |  |  |  |  |  |  |  |  |  |  |  |
| 131.0815 | 16.3 | Amino-proline |  |  |  |  |  |  |  |  |  |  |  |
| 268.1038 | 12.9 | adenosine |  |  |  |  |  |  |  |  |  |  |  |
| 238.0934 | 12.0 | biopterin |  |  |  |  |  |  |  |  |  |  |  |
| 98.98419 | 17.0 | Phosphoric acid |  |  |  |  |  |  |  |  |  |  |  |
| 330.2272 | 6.0 | 6-Keto-decanoylcarnitine |  |  |  |  |  |  |  |  |  |  |  |
| 147.1128 | 26.4 | lysine |  |  |  |  |  |  |  |  |  |  |  |
| 218.1386 | 13.1 | Propionyl-L-carnitine |  |  |  |  |  |  |  |  |  |  |  |
| 245.1605 | 14.4 | Ethyl-Acetyl-Arginine |  |  |  |  |  |  |  |  |  |  |  |
| 180.0515 | 11.2 | xanthopterin |  |  |  |  |  |  |  |  |  |  |  |
| 398.3261 | 10.5 | 9-Hexadecenoylcarnitine |  |  |  |  |  |  |  |  |  |  |  |
| 238.0933 | 9.4 | biopterin |  |  |  |  |  |  |  |  |  |  |  |
| 412.3054 | 5.9 | 3-Hydroxyhexadecadienoylcarnitine |  |  |  |  |  |  |  |  |  |  |  |
| 400.3418 | 10.4 | [FA] O-Palmitoyl-R-carnitine |  |  |  |  |  |  |  |  |  |  |  |
| 217.1296 | 16.2 | Acetyl-arginine |  |  |  |  |  |  |  |  |  |  |  |
| 298.0966 | 11.6 | 5'-Methylthioadenosine |  |  |  |  |  |  |  |  |  |  |  |
| 167.0565 | 16.6 | 1-Methylxanthine |  |  |  |  |  |  |  |  |  |  |  |
| 131.0814 | 17.4 | Amino-proline |  |  |  |  |  |  |  |  |  |  |  |
| 664.1163 | 21.1 | NAD+ |  |  |  |  |  |  |  |  |  |  |  |
| 426.3576 | 10.4 | 11Z-Octadecenylcarnitine |  |  |  |  |  |  |  |  |  |  |  |
| 240.1091 | 13.7 | 6-Lactoyl-5,6,7,8-tetrahydropterin |  |  |  |  |  |  |  |  |  |  |  |
| 182.0812 | 14.4 | D-Tyrosine |  |  |  |  |  |  |  |  |  |  |  |
| 424.0773 | 11.7 | Xanthommatin |  |  |  |  |  |  |  |  |  |  |  |
| 261.037 | 17.0 | hexose phosphate |  |  |  |  |  |  |  |  |  |  |  |
| 164.0567 | 11.3 | Pterin |  |  |  |  |  |  |  |  |  |  |  |
| 176.0553 | 8.5 | N-Formyl-L-glutamic acid |  |  |  |  |  |  |  |  |  |  |  |
| 240.109 | 12.9 | isomer 6-Lactoyl-5,6,7,8-tetrahydropterin |  |  |  |  |  |  |  |  |  |  |  |
| 130.0862 | 14.8 | methyl proline |  |  |  |  |  |  |  |  |  |  |  |
| 124.0393 | 8.5 | Nicotinic acid |  |  |  |  |  |  |  |  |  |  |  |
| 424.3418 | 10.4 | Linolenylcarnitine |  |  |  |  |  |  |  |  |  |  |  |
| 261.0369 | 15.7 | hexose phosphate |  |  |  |  |  |  |  |  |  |  |  |
| 137.0458 | 10.0 | hypoxanthine |  |  |  |  |  |  |  |  |  |  |  |
| 384.2742 | 5.9 | 3-Hydroxy-5, 8-tetradecadiencarnitine |  |  |  |  |  |  |  |  |  |  |  |
| 237.0869 | 12.3 | N-Formylkynurenine |  |  |  |  |  |  |  |  |  |  |  |
| 241.031 | 20.3 | L-Cystine |  |  |  |  |  |  |  |  |  |  |  |
| 372.3104 | 10.5 | Tetradecanoylcarnitine |  |  |  |  |  |  |  |  |  |  |  |
| 349.0541 | 14.8 | Inosine 2'-phosphate |  |  |  |  |  |  |  |  |  |  |  |
| 316.2118 | 6.0 | ketononaoylcarntine |  |  |  |  |  |  |  |  |  |  |  |
| 220.1179 | 7.4 | Pantothenic acid |  |  |  |  |  |  |  |  |  |  |  |
| 364.0649 | 16.5 | GMP |  |  |  |  |  |  |  |  |  |  |  |
| 104.0706 | 15.1 | aminobutyric acid |  |  |  |  |  |  |  |  |  |  |  |
| 162.0761 | 13.6 | Aminoadipic acid |  |  |  |  |  |  |  |  |  |  |  |
| 111.0553 | 17.1 | Imidazole-4-acetaldehyde |  |  |  |  |  |  |  |  |  |  |  |
| 386.2898 | 5.9 | 3-Hydroxy-cis-5-tetradecenoylcarnitine |  |  |  |  |  |  |  |  |  |  |  |
| 159.0513 | 13.1 | (R)(-)-Allantoin |  |  |  |  |  |  |  |  |  |  |  |
| 456.4047 | 10.2 | Arachidyl carnitine |  |  |  |  |  |  |  |  |  |  |  |
| 188.1758 | 34.5 | N-acetylspermidine |  |  |  |  |  |  |  |  |  |  |  |
| 146.1175 | 14.7 | 3-Dehydroxycarnitine |  |  |  |  |  |  |  |  |  |  |  |
| 134.0447 | 16.4 | L-Aspartic acid |  |  |  |  |  |  |  |  |  |  |  |
| 243.0264 | 15.7 | Inositol cyclic phosphate |  |  |  |  |  |  |  |  |  |  |  |
| 282.1195 | 16.8 | 3'-O-Methyladenosine |  |  |  |  |  |  |  |  |  |  |  |
| 150.0775 | 15.8 | Methyladenine |  |  |  |  |  |  |  |  |  |  |  |
| 261.0369 | 17.8 | hexose phosphate |  |  |  |  |  |  |  |  |  |  |  |
| 268.1039 | 11.5 | Deoxyguanosine |  |  |  |  |  |  |  |  |  |  |  |
| 189.087 | 16.0 | N-Acetylglutamine |  |  |  |  |  |  |  |  |  |  |  |
| 428.3733 | 10.3 | Stearoylcarnitine |  |  |  |  |  |  |  |  |  |  |  |
| 182.0673 | 11.2 | Dihydroxanthopterin |  |  |  |  |  |  |  |  |  |  |  |
| 161.0921 | 16.4 | N5-Methyl-L-glutamine |  |  |  |  |  |  |  |  |  |  |  |
| 236.0777 | 9.2 | Dehydrosepiapterin |  |  |  |  |  |  |  |  |  |  |  |
| 243.0265 | 17.0 | Inositol cyclic phosphate |  |  |  |  |  |  |  |  |  |  |  |
| 325.043 | 14.2 | Uridine 2'-phosphate |  |  |  |  |  |  |  |  |  |  |  |
| 144.102 | 14.9 | dimethyl proline |  |  |  |  |  |  |  |  |  |  |  |
| 133.0971 | 26.5 | D-Ornithine |  |  |  |  |  |  |  |  |  |  |  |
| 176.0918 | 13.4 | N-Carboxyethyl-g-aminobutyric acid |  |  |  |  |  |  |  |  |  |  |  |
| 153.0407 | 9.6 | Xanthine |  |  |  |  |  |  |  |  |  |  |  |
| 180.0517 | 12.1 | Isoxanthopterin |  |  |  |  |  |  |  |  |  |  |  |
| 203.1503 | 23.1 | Dimethyl-L-arginine |  |  |  |  |  |  |  |  |  |  |  |
| 118.0611 | 14.6 | Guanidoacetic acid |  |  |  |  |  |  |  |  |  |  |  |
| 229.1548 | 12.7 | L-leucyl-L-proline |  |  |  |  |  |  |  |  |  |  |  |
| 180.088 | 11.6 | 7-Aminomethyl-7-carbaguanine |  |  |  |  |  |  |  |  |  |  |  |
| 175.1078 | 16.0 | N-Acetylornithine |  |  |  |  |  |  |  |  |  |  |  |
| 245.0767 | 9.9 | uridine |  |  |  |  |  |  |  |  |  |  |  |
| 422.3262 | 10.4 | Alpha-linolenyl carnitine |  |  |  |  |  |  |  |  |  |  |  |
| 229.1547 | 13.4 | L-leucyl-L-proline |  |  |  |  |  |  |  |  |  |  |  |
| 196.0829 | 11.1 | 2-Amino-4-hydroxy-6-hydroxymethyl-7,8-dihydropteridine |  |  |  |  |  |  |  |  |  |  |  |
| 447.0676 | 20.0 | CDP-ethanolamine |  |  |  |  |  |  |  |  |  |  |  |
| 213.1234 | 15.0 | L-prolyl-L-proline |  |  |  |  |  |  |  |  |  |  |  |
| 255.0974 | 17.6 | glycyl-dopa |  |  |  |  |  |  |  |  |  |  |  |
| 377.1454 | 8.6 | Riboflavin |  |  |  |  |  |  |  |  |  |  |  |
| 324.059 | 18.3 | Cytidine monophosphate |  |  |  |  |  |  |  |  |  |  |  |
| 160.1332 | 14.9 | DL-2-Aminooctanoic acid |  |  |  |  |  |  |  |  |  |  |  |
| 134.0811 | 16.0 | Hydroxyvaline |  |  |  |  |  |  |  |  |  |  |  |
| 219.0975 | 15.0 | gamma-L-Glutamyl-D-alanine |  |  |  |  |  |  |  |  |  |  |  |
| 176.0918 | 15.1 | N-Carboxyethyl-g-aminobutyric acid |  |  |  |  |  |  |  |  |  |  |  |
| 130.0499 | 7.5 | pyroglutamic acid |  |  |  |  |  |  |  |  |  |  |  |
| 215.1391 | 13.3 | Dethiobiotin |  |  |  |  |  |  |  |  |  |  |  |
| 260.0528 | 15.9 | D-Glucosamine 6-phosphate |  |  |  |  |  |  |  |  |  |  |  |
| 176.103 | 17.6 | citrulline |  |  |  |  |  |  |  |  |  |  |  |
| 254.0883 | 10.5 | Hydroxysepiapterin |  |  |  |  |  |  |  |  |  |  |  |
| 302.0635 | 17.0 | N-Acetyl-D-galactosamine 6-phosphate |  |  |  |  |  |  |  |  |  |  |  |
| 427.095 | 19.7 | S-Glutathionyl-L-cysteine |  |  |  |  |  |  |  |  |  |  |  |
| 298.1144 | 10.3 | 1-Methylguanosine |  |  |  |  |  |  |  |  |  |  |  |
| 143.0816 | 14.2 | Ectoine |  |  |  |  |  |  |  |  |  |  |  |
| 414.321 | 5.8 | 3-Hydroxy-9-hexadecenoylcarnitine |  |  |  |  |  |  |  |  |  |  |  |
| 464.0813 | 13.8 | Adenylsuccinic acid |  |  |  |  |  |  |  |  |  |  |  |
| 113.0345 | 9.9 | Uracil |  |  |  |  |  |  |  |  |  |  |  |
| 205.1184 | 16.4 | N5-(L-1-Carboxyethyl)-L-ornithine |  |  |  |  |  |  |  |  |  |  |  |
| 198.0874 | 14.4 | N-Acetyl-L-histidine |  |  |  |  |  |  |  |  |  |  |  |
| 268.1025 | 18.1 | Neuraminic acid |  |  |  |  |  |  |  |  |  |  |  |
| 123.0552 | 9.9 | nicotinamide |  |  |  |  |  |  |  |  |  |  |  |
| 244.0927 | 18.1 | cytidine |  |  |  |  |  |  |  |  |  |  |  |
| 335.0636 | 17.5 | Nicotinamide D-ribonucleotide |  |  |  |  |  |  |  |  |  |  |  |
| 124.0393 | 7.5 | Nicotinic acid isomer |  |  |  |  |  |  |  |  |  |  |  |
| 133.0607 | 9.3 | asparagine isomer |  |  |  |  |  |  |  |  |  |  |  |
| 168.0519 | 15.4 | 2,8-Dihydroxyadenine |  |  |  |  |  |  |  |  |  |  |  |
| 309.1655 | 27.0 | Fructoselysine |  |  |  |  |  |  |  |  |  |  |  |
| 190.1186 | 27.9 | Homocitrulline |  |  |  |  |  |  |  |  |  |  |  |
| 344.2787 | 10.7 | Dodecanoylcarnitine |  |  |  |  |  |  |  |  |  |  |  |
| 229.1546 | 14.8 | L-leucyl-L-proline |  |  |  |  |  |  |  |  |  |  |  |
| 161.0921 | 15.6 | N5-Methyl-L-glutamine |  |  |  |  |  |  |  |  |  |  |  |
| 127.0502 | 15.7 | Imidazole-4-acetate |  |  |  |  |  |  |  |  |  |  |  |
| 196.083 | 10.0 | 2-Amino-4-hydroxy-6-hydroxymethyl-7,8-dihydropteridine |  |  |  |  |  |  |  |  |  |  |  |
| 136.0618 | 14.8 | Adenine |  |  |  |  |  |  |  |  |  |  |  |
| 112.0506 | 17.7 | Cytosine |  |  |  |  |  |  |  |  |  |  |  |
| 148.0604 | 14.1 | N-(Carboxymethyl)-D-alanine |  |  |  |  |  |  |  |  |  |  |  |
| 370.2947 | 10.6 | trans-2-Tetradecenoylcarnitine |  |  |  |  |  |  |  |  |  |  |  |
| 131.1178 | 17.1 | N-Acetylputrescine |  |  |  |  |  |  |  |  |  |  |  |
| 197.067 | 9.2 | 1,3-Dimethyluric acid |  |  |  |  |  |  |  |  |  |  |  |
| 146.1652 | 40.5 | spermidine |  |  |  |  |  |  |  |  |  |  |  |
| 166.0724 | 14.1 | N2-Methylguanine |  |  |  |  |  |  |  |  |  |  |  |
| 189.1235 | 16.2 | N-Alpha-acetyllysine |  |  |  |  |  |  |  |  |  |  |  |
| 122.0964 | 13.4 | Phenylethylamine |  |  |  |  |  |  |  |  |  |  |  |
| 166.0724 | 18.4 | N2-Methylguanine |  |  |  |  |  |  |  |  |  |  |  |
| 189.1347 | 33.5 | Homoarginine |  |  |  |  |  |  |  |  |  |  |  |
| 139.0502 | 14.2 | Urocanic acid |  |  |  |  |  |  |  |  |  |  |  |
| 140.082 | 24.0 | L-Histidinal |  |  |  |  |  |  |  |  |  |  |  |
| 248.1492 | 14.3 | Hydroxybutyrylcarnitine |  |  |  |  |  |  |  |  |  |  |  |
| 400.1284 | 26.4 | S-inosylmethione |  |  |  |  |  |  |  |  |  |  |  |
| 223.0747 | 20.7 | Allocystathionine |  |  |  |  |  |  |  |  |  |  |  |
| 247.0577 | 13.4 | Glycerophosphoglycerol |  |  |  |  |  |  |  |  |  |  |  |
| 152.0568 | 10.3 | guanine |  |  |  |  |  |  |  |  |  |  |  |
| 388.3054 | 10.8 | 2-Hydroxymyristoylcarnitine |  |  |  |  |  |  |  |  |  |  |  |
| 384.1148 | 9.7 | Succinyladenosine |  |  |  |  |  |  |  |  |  |  |  |
| 196.0969 | 9.6 | 2-Phenyl-1,3-propanediyl monocarbamate |  |  |  |  |  |  |  |  |  |  |  |
| 286.1031 | 9.9 | N4-Acetylcytidine |  |  |  |  |  |  |  |  |  |  |  |
| 414.3575 | 10.4 | Heptadecanoyl carnitine |  |  |  |  |  |  |  |  |  |  |  |
| 442.3515 | 10.6 | 3-Hydroxy-9Z-octadecenoylcarnitine |  |  |  |  |  |  |  |  |  |  |  |
| 173.0921 | 16.9 | L-prolyl-L-glycine |  |  |  |  |  |  |  |  |  |  |  |
| 168.0325 | 14.1 | Homocysteinesulfinic acid |  |  |  |  |  |  |  |  |  |  |  |
| 254.0883 | 14.3 | Hydroxysepiapterin |  |  |  |  |  |  |  |  |  |  |  |
| 146.0924 | 15.4 | 4-Guanidinobutanoic acid |  |  |  |  |  |  |  |  |  |  |  |
| 118.1226 | 16.1 | 2-Methylcholine |  |  |  |  |  |  |  |  |  |  |  |
| 265.1117 | 31.4 | Thiamine |  |  |  |  |  |  |  |  |  |  |  |
| 291.0473 | 16.0 | D-Sedoheptulose 7-phosphate |  |  |  |  |  |  |  |  |  |  |  |
| 613.1591 | 19.3 | Oxidized glutathione |  |  |  |  |  |  |  |  |  |  |  |
| 89.10735 | 36.2 | putrescine |  |  |  |  |  |  |  |  |  |  |  |
| 136.0615 | 10.8 | Adenine isomer |  |  |  |  |  |  |  |  |  |  |  |
| 256.1039 | 11.8 | 7,8-Dihydroneopterin |  |  |  |  |  |  |  |  |  |  |  |
| 160.1332 | 11.3 | DL-2-Aminooctanoic acid |  |  |  |  |  |  |  |  |  |  |  |
| 184.083 | 17.2 | 2,6-Diamino-4-hydroxy-5-N-methylformamidopyrimidine |  |  |  |  |  |  |  |  |  |  |  |
| 399.3255 | 5.9 | (5Z,7E,22E)-(1S,3R)-9,10-seco-5,7,10(19),22-cholestatetraene-1,3-diol |  |  |  |  |  |  |  |  |  |  |  |
| 196.0466 | 12.7 | hydromethyldeoxyhydropterin |  |  |  |  |  |  |  |  |  |  |  |
| 175.119 | 28.6 | L-Arginine |  |  |  |  |  |  |  |  |  |  |  |
| 191.1027 | 21.9 | Diaminopimelic acid |  |  |  |  |  |  |  |  |  |  |  |
| 189.1597 | 27.6 | trimethyllysine |  |  |  |  |  |  |  |  |  |  |  |
| 196.083 | 8.6 | 2-Amino-4-hydroxy-6-hydroxymethyl-7,8-dihydropteridine |  |  |  |  |  |  |  |  |  |  |  |
| 291.1298 | 22.3 | Argininosuccinic acid |  |  |  |  |  |  |  |  |  |  |  |
| 288.2167 | 6.0 | L-Octanoylcarnitine |  |  |  |  |  |  |  |  |  |  |  |
| 355.1545 | 40.1 | decarboxy S-adenosylmethionine |  |  |  |  |  |  |  |  |  |  |  |
| 253.0931 | 10.0 | N-D-Ribosylpurine |  |  |  |  |  |  |  |  |  |  |  |
| 171.0514 | 9.3 | 5-Ureido-4-imidazole carboxylate |  |  |  |  |  |  |  |  |  |  |  |
| 255.0852 | 21.8 | phosphoarginine |  |  |  |  |  |  |  |  |  |  |  |
| 174.0874 | 17.1 | Oxoarginine |  |  |  |  |  |  |  |  |  |  |  |
| 180.0866 | 17.7 | hexosamine |  |  |  |  |  |  |  |  |  |  |  |
| 314.2325 | 6.0 | 9-Decenoylcarnitine |  |  |  |  |  |  |  |  |  |  |  |
| 237.1094 | 11.1 | S-aminomethyldihydrolipoamide |  |  |  |  |  |  |  |  |  |  |  |
| 396.3106 | 10.6 | 9,12-Hexadecadienoylcarnitine |  |  |  |  |  |  |  |  |  |  |  |
| 189.1347 | 23.8 | Homoarginine |  |  |  |  |  |  |  |  |  |  |  |
| 302.2325 | 6.0 | Nonanoylcarnitine |  |  |  |  |  |  |  |  |  |  |  |
| 183.0513 | 11.2 | 7-Methyluric acid |  |  |  |  |  |  |  |  |  |  |  |
| 171.0053 | 15.0 | D-Glyceraldehyde 3-phosphate |  |  |  |  |  |  |  |  |  |  |  |
| 174.0551 | 12.6 | Quinaldic acid |  |  |  |  |  |  |  |  |  |  |  |
| 175.0715 | 16.4 | N-Acetylasparagine |  |  |  |  |  |  |  |  |  |  |  |
| 245.0767 | 11.5 | Pseudouridine |  |  |  |  |  |  |  |  |  |  |  |
| 198.0874 | 17.1 | N-Acetyl-L-histidine isomer |  |  |  |  |  |  |  |  |  |  |  |
| 256.0815 | 14.4 | Nicotinate D-ribonucleoside |  |  |  |  |  |  |  |  |  |  |  |
| 199.0826 | 13.3 | 5-Acetylamino-6-amino-3-methyluracil |  |  |  |  |  |  |  |  |  |  |  |
| 180.088 | 12.9 | 7-Aminomethyl-7-carbaguanine |  |  |  |  |  |  |  |  |  |  |  |
| 242.1134 | 11.8 | 5-Methyldeoxycytidine |  |  |  |  |  |  |  |  |  |  |  |
| 161.1285 | 25.4 | methyllysine |  |  |  |  |  |  |  |  |  |  |  |
| 276.1805 | 13.3 | Hydroxyhexanoycarnitine |  |  |  |  |  |  |  |  |  |  |  |
| 243.0876 | 6.1 | Lumichrome |  |  |  |  |  |  |  |  |  |  |  |
| 540.4978 | 10.0 | Hexacosanoyl carnitine |  |  |  |  |  |  |  |  |  |  |  |
| 312.2168 | 6.0 | 2-trans,4-cis-Decadienoylcarnitine |  |  |  |  |  |  |  |  |  |  |  |
| 221.092 | 13.6 | 5-Hydroxytryptophan |  |  |  |  |  |  |  |  |  |  |  |
| 360.2742 | 11.1 | 2-Hydroxylauroylcarnitine |  |  |  |  |  |  |  |  |  |  |  |
| 399.1443 | 33.1 | S-adenosylmethionine |  |  |  |  |  |  |  |  |  |  |  |
| 302.2325 | 11.6 | Nonanoylcarnitine |  |  |  |  |  |  |  |  |  |  |  |
| 138.0914 | 16.4 | tyramine |  |  |  |  |  |  |  |  |  |  |  |
| 368.2787 | 10.6 | 3, 5-Tetradecadiencarnitine |  |  |  |  |  |  |  |  |  |  |  |
| 308.0909 | 14.9 | Glutathione |  |  |  |  |  |  |  |  |  |  |  |
| 342.263 | 10.7 | trans-2-Dodecenoylcarnitine |  |  |  |  |  |  |  |  |  |  |  |
| 274.2011 | 11.7 | Heptanoylcarnitine |  |  |  |  |  |  |  |  |  |  |  |
| 268.0849 | 16.8 | S-Ribosyl-L-homocysteine |  |  |  |  |  |  |  |  |  |  |  |
| 198.0761 | 16.8 | DL-Dopa |  |  |  |  |  |  |  |  |  |  |  |
| 251.0695 | 14.4 | glutamylcysteine |  |  |  |  |  |  |  |  |  |  |  |
| 260.1856 | 11.6 | L-Hexanoylcarnitine |  |  |  |  |  |  |  |  |  |  |  |
| 122.027 | 15.2 | L-Cysteine |  |  |  |  |  |  |  |  |  |  |  |
| 288.2168 | 11.2 | L-Octanoylcarnitine |  |  |  |  |  |  |  |  |  |  |  |
| 316.2481 | 10.9 | O-Decanoyl-L-carnitine |  |  |  |  |  |  |  |  |  |  |  |
| 356.1384 | 37.4 | decarboxy S-adenosylinosylmethionine |  |  |  |  |  |  |  |  |  |  |  |
| 338.1014 | 15.4 | S-(Hydroxymethyl)glutathione |  |  |  |  |  |  |  |  |  |  |  |
