# Supplementary material for: Mapping an Atlas of Tissue-Specific Drosophila melanogaster Metabolomes by High Resolution Mass Spectrometry
Source: PLoS One. 2013 Oct 29;8(10):e78066. doi: 10.1371/journal.pone.0078066 (PMC3812166; doi:10.1371/journal.pone.0078066)
Supplement: Table S4 — Positively charged lipids in Drosophila ranked according to abundance in the whole fly. (DOCX) [file pone.0078066.s004.docx]

**Table S4** Positively charged lipids in Drosophila ranked according to abundance in the whole fly.

| M/Z | RT | **Name** | WF | HD | Crop | MG | AT | PT | HG | Ov | Test | ACCG | CUT |
| --- | --- | --- | --- | --- | --- | --- | --- | --- | --- | --- | --- | --- | --- |
| 520.3397 | 21.4 | Lyso PC 18:2 |  |  |  |  |  |  |  |  |  |  |  |
| 494.324 | 21.5 | Lyso PC 16:1 |  |  |  |  |  |  |  |  |  |  |  |
| 758.569 | 19.5 | PC 34:2 |  |  |  |  |  |  |  |  |  |  |  |
| 756.554 | 19.4 | PC 34:3 |  |  |  |  |  |  |  |  |  |  |  |
| 782.5693 | 19.3 | PC 36:4 |  |  |  |  |  |  |  |  |  |  |  |
| 522.3551 | 21.4 | Lyso PC 18:1 |  |  |  |  |  |  |  |  |  |  |  |
| 480.3086 | 14.6 | Lyso PE 18:1 |  |  |  |  |  |  |  |  |  |  |  |
| 480.3083 | 13.3 | Lyso PE 18:1 |  |  |  |  |  |  |  |  |  |  |  |
| 478.2929 | 14.6 | Lyso PE 18:2 |  |  |  |  |  |  |  |  |  |  |  |
| 478.2927 | 13.3 | Lyso PE 18:2 |  |  |  |  |  |  |  |  |  |  |  |
| 784.5842 | 19.3 | PC 36:3 |  |  |  |  |  |  |  |  |  |  |  |
| 732.5533 | 19.6 | PC 32:1 |  |  |  |  |  |  |  |  |  |  |  |
| 452.2771 | 14.7 | Lyso PE 16:1 |  |  |  |  |  |  |  |  |  |  |  |
| 452.2766 | 13.4 | Lyso PE 16:1 |  |  |  |  |  |  |  |  |  |  |  |
| 730.5375 | 19.5 | PC 34:2 |  |  |  |  |  |  |  |  |  |  |  |
| 760.5843 | 19.6 | PC 34:1 |  |  |  |  |  |  |  |  |  |  |  |
| 756.5542 | 20.7 | PC 34:3 |  |  |  |  |  |  |  |  |  |  |  |
| 742.5374 | 12.0 | PE 36:3 |  |  |  |  |  |  |  |  |  |  |  |
| 518.3241 | 21.5 | Lyso PC 18:3 |  |  |  |  |  |  |  |  |  |  |  |
| 740.522 | 12.0 | PE 36:4 |  |  |  |  |  |  |  |  |  |  |  |
| 716.5222 | 12.1 | PE 34:2 |  |  |  |  |  |  |  |  |  |  |  |
| 784.5857 | 20.7 | PC 36:3 |  |  |  |  |  |  |  |  |  |  |  |
| 730.5372 | 20.8 | PC 32:2 |  |  |  |  |  |  |  |  |  |  |  |
| 786.5992 | 19.4 | PC 36:2 |  |  |  |  |  |  |  |  |  |  |  |
| 714.507 | 12.1 | PE 34:3 |  |  |  |  |  |  |  |  |  |  |  |
| 476.2773 | 14.6 | Lyso PE 18:3 |  |  |  |  |  |  |  |  |  |  |  |
| 718.5376 | 12.2 | PE 34:1 |  |  |  |  |  |  |  |  |  |  |  |
| 476.2773 | 13.3 | Lyso PE 18.3 |  |  |  |  |  |  |  |  |  |  |  |
| 728.5584 | 12.2 | PE ether 36:2 |  |  |  |  |  |  |  |  |  |  |  |
| 496.34 | 21.4 | Lyso PC 16:0 |  |  |  |  |  |  |  |  |  |  |  |
| 782.5698 | 20.7 | PC 36:4 |  |  |  |  |  |  |  |  |  |  |  |
| 690.5066 | 12.2 | PE 32:1 |  |  |  |  |  |  |  |  |  |  |  |
| 744.5516 | 12.0 | PE 36:2 |  |  |  |  |  |  |  |  |  |  |  |
| 754.5383 | 19.4 | PC 34:4 |  |  |  |  |  |  |  |  |  |  |  |
| 794.7215 | 2.8 | TG 46:0 |  |  |  |  |  |  |  |  |  |  |  |
| 704.5223 | 19.7 | PE ether 34:0 |  |  |  |  |  |  |  |  |  |  |  |
| 766.6904 | 2.8 | TG 44:0 |  |  |  |  |  |  |  |  |  |  |  |
| 792.7068 | 2.7 | TG 46:1 |  |  |  |  |  |  |  |  |  |  |  |
| 689.5592 | 13.1 | SM 36:1 PE |  |  |  |  |  |  |  |  |  |  |  |
| 820.7372 | 2.8 | TG 48:1 |  |  |  |  |  |  |  |  |  |  |  |
| 734.5698 | 19.8 | PC 32:0 |  |  |  |  |  |  |  |  |  |  |  |
| 738.5067 | 11.9 | PE 36:5 |  |  |  |  |  |  |  |  |  |  |  |
| 687.5439 | 13.1 | SM PE 36:2 |  |  |  |  |  |  |  |  |  |  |  |
| 524.2985 | 13.3 | Lyso PS 18:0 |  |  |  |  |  |  |  |  |  |  |  |
| 661.5281 | 13.1 | SM PE 34:1 |  |  |  |  |  |  |  |  |  |  |  |
| 468.3084 | 21.5 | Lyso PC 14:0 |  |  |  |  |  |  |  |  |  |  |  |
| 659.5124 | 13.1 | SM PE 34:2 |  |  |  |  |  |  |  |  |  |  |  |
| 688.4908 | 12.2 | PE 32:2 |  |  |  |  |  |  |  |  |  |  |  |
| 788.6158 | 19.6 | PC 36:1 |  |  |  |  |  |  |  |  |  |  |  |
| 764.6757 | 2.7 | TG 44:1 |  |  |  |  |  |  |  |  |  |  |  |
| 818.7228 | 2.7 | TG 48:2 |  |  |  |  |  |  |  |  |  |  |  |
| 728.5229 | 19.6 | PC 32:3 |  |  |  |  |  |  |  |  |  |  |  |
| 288.2896 | 11.0 | Sphinganine 17:0 |  |  |  |  |  |  |  |  |  |  |  |
| 788.5436 | 11.7 | PS 36:2 |  |  |  |  |  |  |  |  |  |  |  |
| 848.7687 | 2.9 | TG 50:1 |  |  |  |  |  |  |  |  |  |  |  |
| 454.2928 | 13.5 | Lyso PE 16:0 |  |  |  |  |  |  |  |  |  |  |  |
| 704.5216 | 21.0 | PC 30:1 |  |  |  |  |  |  |  |  |  |  |  |
| 706.5386 | 19.8 | PC 30:0 |  |  |  |  |  |  |  |  |  |  |  |
| 772.5845 | 19.5 | PC 36:9 |  |  |  |  |  |  |  |  |  |  |  |
| 730.5751 | 12.2 | PE ether 36:1 |  |  |  |  |  |  |  |  |  |  |  |
| 786.528 | 11.7 | PS 36:3 |  |  |  |  |  |  |  |  |  |  |  |
| 780.48 | 12.1 | PS 36:6 |  |  |  |  |  |  |  |  |  |  |  |
| 566.5505 | 2.9 | Cer 36:1 |  |  |  |  |  |  |  |  |  |  |  |
| 846.7541 | 2.8 | TG 50:2 |  |  |  |  |  |  |  |  |  |  |  |
| 364.2594 | 13.5 | Hist C14:1 |  |  |  |  |  |  |  |  |  |  |  |
| 538.5193 | 2.9 | Cer 34:0 |  |  |  |  |  |  |  |  |  |  |  |
| 780.4808 | 11.7 | PS 36:6 |  |  |  |  |  |  |  |  |  |  |  |
| 742.5731 | 19.5 | PC ether 34:5 |  |  |  |  |  |  |  |  |  |  |  |
| 716.5231 | 13.4 | PE 34:2 |  |  |  |  |  |  |  |  |  |  |  |
| 712.4912 | 12.1 | PE 34:4 |  |  |  |  |  |  |  |  |  |  |  |
| 746.5685 | 12.1 | PE 36:1 |  |  |  |  |  |  |  |  |  |  |  |
| 288.2894 | 12.5 | Sphinganine 17:0 |  |  |  |  |  |  |  |  |  |  |  |
| 784.5122 | 11.7 | PS 36:4 |  |  |  |  |  |  |  |  |  |  |  |
| 844.7383 | 2.8 | TG 50:3 |  |  |  |  |  |  |  |  |  |  |  |
| 744.5909 | 19.5 | PC ether 34:1 |  |  |  |  |  |  |  |  |  |  |  |
| 736.4908 | 11.9 | PE 36:6 |  |  |  |  |  |  |  |  |  |  |  |
| 732.5908 | 12.3 | PE ether 36:0 |  |  |  |  |  |  |  |  |  |  |  |
| 816.7073 | 2.8 | TG 48:3 |  |  |  |  |  |  |  |  |  |  |  |
| 678.5071 | 19.8 | PC 28:0 |  |  |  |  |  |  |  |  |  |  |  |
| 714.507 | 13.4 | PE 34:3 |  |  |  |  |  |  |  |  |  |  |  |
| 715.5748 | 13.3 | SM PE 38:2 |  |  |  |  |  |  |  |  |  |  |  |
| 703.5382 | 13.4 | SM PE 36:2 hydroxy |  |  |  |  |  |  |  |  |  |  |  |
| 774.6003 | 19.5 | PC 36:8 |  |  |  |  |  |  |  |  |  |  |  |
| 702.506 | 19.6 | PC 30:2 |  |  |  |  |  |  |  |  |  |  |  |
| 662.4752 | 12.3 | PE 30:1 |  |  |  |  |  |  |  |  |  |  |  |
| 440.2768 | 21.7 | Lyso PC 12:0 |  |  |  |  |  |  |  |  |  |  |  |
| 466.293 | 13.3 | Lyso PC 14:0 |  |  |  |  |  |  |  |  |  |  |  |
| 466.2925 | 21.6 | Lyso PC 14:0 |  |  |  |  |  |  |  |  |  |  |  |
| 717.59 | 13.2 | SM PE 38:1 |  |  |  |  |  |  |  |  |  |  |  |
| 756.5897 | 12.2 | PE ether 38:2 |  |  |  |  |  |  |  |  |  |  |  |
| 782.4967 | 11.7 | PS 36:5 |  |  |  |  |  |  |  |  |  |  |  |
| 633.4969 | 13.4 | SM PE 32:1 |  |  |  |  |  |  |  |  |  |  |  |
| 426.2614 | 13.4 | Lyso PE 14:0 |  |  |  |  |  |  |  |  |  |  |  |
| 676.4907 | 19.8 | PC 28:1 |  |  |  |  |  |  |  |  |  |  |  |
| 770.6066 | 19.7 | PC ether 36:2 |  |  |  |  |  |  |  |  |  |  |  |
| 716.5667 | 3.0 | GC 34:1 hydroxy |  |  |  |  |  |  |  |  |  |  |  |
| 316.3207 | 12.3 | Sphinganine 19:0 |  |  |  |  |  |  |  |  |  |  |  |
| 288.2895 | 9.6 | Sphinganine 17:0 |  |  |  |  |  |  |  |  |  |  |  |
| 874.7851 | 2.9 | TG 52:2 |  |  |  |  |  |  |  |  |  |  |  |
| 872.7698 | 2.9 | TG 52:3 |  |  |  |  |  |  |  |  |  |  |  |
| 760.5123 | 11.7 | PS 34:2 |  |  |  |  |  |  |  |  |  |  |  |
| 704.5592 | 12.3 | PE ether 34:0 |  |  |  |  |  |  |  |  |  |  |  |
| 700.5721 | 3.0 | GC 34:1 |  |  |  |  |  |  |  |  |  |  |  |
| 664.4918 | 12.3 | PE 30:0 |  |  |  |  |  |  |  |  |  |  |  |
| 726.5434 | 11.9 | PE ether 36:3 |  |  |  |  |  |  |  |  |  |  |  |
| 772.6218 | 19.8 | PC 36:1 |  |  |  |  |  |  |  |  |  |  |  |
| 700.5278 | 12.1 | PE ether 34.2 |  |  |  |  |  |  |  |  |  |  |  |
| 352.3207 | 3.1 | ET C20:2 |  |  |  |  |  |  |  |  |  |  |  |
| 758.6057 | 12.3 | PE ether 38:1 |  |  |  |  |  |  |  |  |  |  |  |
| 876.8004 | 3.0 | TG 52:1 |  |  |  |  |  |  |  |  |  |  |  |
| 842.7222 | 2.8 | TG 50:4 |  |  |  |  |  |  |  |  |  |  |  |
| 762.5281 | 11.7 | PS 34:1 |  |  |  |  |  |  |  |  |  |  |  |
| 702.5423 | 12.1 | PE ether 34:1 |  |  |  |  |  |  |  |  |  |  |  |
| 506.3243 | 13.3 | Lyso PE 20:2 |  |  |  |  |  |  |  |  |  |  |  |
| 691.5654 | 13.1 | SM 36:0 PE |  |  |  |  |  |  |  |  |  |  |  |
| 594.5821 | 2.8 | SP 38:1 |  |  |  |  |  |  |  |  |  |  |  |
| 768.5902 | 19.3 | PC ether 36:3 |  |  |  |  |  |  |  |  |  |  |  |
| 354.3364 | 3.1 | ET C20:0 |  |  |  |  |  |  |  |  |  |  |  |
| 740.5591 | 19.4 | PC ether 34:3 |  |  |  |  |  |  |  |  |  |  |  |
| 631.4804 | 13.3 | SM PE 32:2 |  |  |  |  |  |  |  |  |  |  |  |
| 650.4748 | 20.0 | PC 26:0 |  |  |  |  |  |  |  |  |  |  |  |
| 686.4757 | 12.1 | PE 32:3 |  |  |  |  |  |  |  |  |  |  |  |
| 316.3209 | 9.5 | Sphinganine 19:0 |  |  |  |  |  |  |  |  |  |  |  |
| 728.6033 | 3.1 | GC 36:1 |  |  |  |  |  |  |  |  |  |  |  |
| 870.7537 | 2.8 | TG 52:4 |  |  |  |  |  |  |  |  |  |  |  |
| 896.7692 | 2.8 | TG 54:5 |  |  |  |  |  |  |  |  |  |  |  |
| 745.5009 | 4.8 | PG(18:3(9Z,12Z,15Z)/16:0) |  |  |  |  |  |  |  |  |  |  |  |
| 804.5518 | 19.3 | PC 38:7 |  |  |  |  |  |  |  |  |  |  |  |
| 724.5717 | 3.0 | GC 36:3 |  |  |  |  |  |  |  |  |  |  |  |
| 814.6912 | 2.8 | TG 48:4 |  |  |  |  |  |  |  |  |  |  |  |
| 663.534 | 13.1 | SM PE 34:0 |  |  |  |  |  |  |  |  |  |  |  |
| 548.371 | 21.3 | Lyso PC 20:2 |  |  |  |  |  |  |  |  |  |  |  |
| 411.3253 | 2.8 | beta-Tocotrienol |  |  |  |  |  |  |  |  |  |  |  |
| 898.7849 | 2.8 | TG 54:4 |  |  |  |  |  |  |  |  |  |  |  |
| 482.4566 | 2.8 | Sphingenine 30:1 |  |  |  |  |  |  |  |  |  |  |  |
| 758.4966 | 11.7 | PS 34:3 |  |  |  |  |  |  |  |  |  |  |  |
| 814.6317 | 19.4 | PC 38:2 |  |  |  |  |  |  |  |  |  |  |  |
| 636.4603 | 12.3 | PE 28:0 |  |  |  |  |  |  |  |  |  |  |  |
| 760.6221 | 12.3 | PE 38:2 |  |  |  |  |  |  |  |  |  |  |  |
| 508.3401 | 13.5 | Lyso PE 20:1 |  |  |  |  |  |  |  |  |  |  |  |
| 696.5412 | 3.1 | GC 34:3 |  |  |  |  |  |  |  |  |  |  |  |
| 812.6151 | 19.4 | PC 38:3 |  |  |  |  |  |  |  |  |  |  |  |
| 744.5986 | 3.0 | GC 36:1 hydroxy |  |  |  |  |  |  |  |  |  |  |  |
| 701.5595 | 13.1 | SM PE 37:2 |  |  |  |  |  |  |  |  |  |  |  |
| 770.5688 | 11.9 | PE 38:3 |  |  |  |  |  |  |  |  |  |  |  |
| 764.5202 | 12.0 | PE 38:6 |  |  |  |  |  |  |  |  |  |  |  |
| 302.3048 | 12.4 | Sphingosine 18:0 |  |  |  |  |  |  |  |  |  |  |  |
| 900.8005 | 2.9 | TG 54:3 |  |  |  |  |  |  |  |  |  |  |  |
| 698.5565 | 3.0 | GC 34:2 |  |  |  |  |  |  |  |  |  |  |  |
| 648.4602 | 20.0 | PC 26:1 |  |  |  |  |  |  |  |  |  |  |  |
| 464.2773 | 21.5 | Lyso PC 14:1 |  |  |  |  |  |  |  |  |  |  |  |
| 338.2434 | 14.6 | Hist 12:0 |  |  |  |  |  |  |  |  |  |  |  |
| 510.4882 | 2.9 | SP 32:2 |  |  |  |  |  |  |  |  |  |  |  |
| 424.2451 | 13.5 | Lyso PE 14:1 |  |  |  |  |  |  |  |  |  |  |  |
| 564.5348 | 2.9 | Spingenine 36:2 |  |  |  |  |  |  |  |  |  |  |  |
| 675.5439 | 13.1 | SM PE 32:1 deoxy |  |  |  |  |  |  |  |  |  |  |  |
| 713.5599 | 13.0 | SM PE 38:3 |  |  |  |  |  |  |  |  |  |  |  |
| 816.6475 | 19.6 | PC 38:1 |  |  |  |  |  |  |  |  |  |  |  |
| 718.5839 | 3.1 | GC 34:0 hydroxy |  |  |  |  |  |  |  |  |  |  |  |
| 698.5107 | 12.0 | PE ether 34:3 |  |  |  |  |  |  |  |  |  |  |  |
| 700.4907 | 19.5 | PC 30:3 |  |  |  |  |  |  |  |  |  |  |  |
| 894.7544 | 2.8 | TG 54:6 |  |  |  |  |  |  |  |  |  |  |  |
| 674.4761 | 19.5 | PC 28:2 |  |  |  |  |  |  |  |  |  |  |  |
| 814.5598 | 11.7 | PS 38:3 |  |  |  |  |  |  |  |  |  |  |  |
| 716.5582 | 19.6 | PC ether 32:2 |  |  |  |  |  |  |  |  |  |  |  |
| 774.5999 | 12.1 | PE 38:1 |  |  |  |  |  |  |  |  |  |  |  |
| 752.6029 | 3.0 | GC 38:3 |  |  |  |  |  |  |  |  |  |  |  |
| 766.5738 | 19.3 | PC ether 36:7 |  |  |  |  |  |  |  |  |  |  |  |
| 718.5753 | 19.8 | PC ether 32:1 |  |  |  |  |  |  |  |  |  |  |  |
| 634.4431 | 12.4 | PE 28:1 |  |  |  |  |  |  |  |  |  |  |  |
| 734.4966 | 11.8 | PS(18:1(9Z)/14:0) |  |  |  |  |  |  |  |  |  |  |  |
| 608.4283 | 12.5 | PE 26:0 |  |  |  |  |  |  |  |  |  |  |  |
| 798.6358 | 19.4 | PC ether 38:3 |  |  |  |  |  |  |  |  |  |  |  |
| 689.5228 | 13.4 | SM PE 32:2 deoxy |  |  |  |  |  |  |  |  |  |  |  |
| 556.5294 | 2.7 | Cer 34:0 hydroxy |  |  |  |  |  |  |  |  |  |  |  |
| 810.5262 | 11.7 | PS 38:5 |  |  |  |  |  |  |  |  |  |  |  |
| 676.5278 | 12.3 | PE ether 32:2 |  |  |  |  |  |  |  |  |  |  |  |
| 304.2844 | 13.2 | Phytosphingolipid |  |  |  |  |  |  |  |  |  |  |  |
| 752.5577 | 11.9 | PE ether 38:4 |  |  |  |  |  |  |  |  |  |  |  |
| 756.6345 | 3.0 | GC 38:1 |  |  |  |  |  |  |  |  |  |  |  |
| 766.5364 | 12.0 | PE 38:5 |  |  |  |  |  |  |  |  |  |  |  |
| 808.5108 | 11.7 | PS 38:6 |  |  |  |  |  |  |  |  |  |  |  |
| 795.6276 | 2.7 | Ubiquinone 9 |  |  |  |  |  |  |  |  |  |  |  |
| 622.4446 | 20.1 | PC 24:0 |  |  |  |  |  |  |  |  |  |  |  |
| 717.5541 | 13.3 | SM 37:2 hydroxy |  |  |  |  |  |  |  |  |  |  |  |
| 498.2835 | 13.1 | Lyso PS 16:0 |  |  |  |  |  |  |  |  |  |  |  |
| 300.2897 | 7.4 | Sphingosine |  |  |  |  |  |  |  |  |  |  |  |
| 672.5407 | 3.0 | GC 32:1 |  |  |  |  |  |  |  |  |  |  |  |
| 660.4601 | 12.3 | PE 30:2 |  |  |  |  |  |  |  |  |  |  |  |
| 726.5883 | 2.9 | GC 36:2 |  |  |  |  |  |  |  |  |  |  |  |
| 447.0671 | 19.7 | CDP-ethanolamine |  |  |  |  |  |  |  |  |  |  |  |
| 706.5381 | 18.5 | PC 30:0 |  |  |  |  |  |  |  |  |  |  |  |
| 550.3866 | 21.4 | Lyso PC 20:1 |  |  |  |  |  |  |  |  |  |  |  |
| 692.5233 | 10.8 | PE 32:0 |  |  |  |  |  |  |  |  |  |  |  |
| 862.6249 | 3.4 | GBC 34:1 |  |  |  |  |  |  |  |  |  |  |  |
| 808.5827 | 19.4 | PC 38:5 |  |  |  |  |  |  |  |  |  |  |  |
| 806.4945 | 11.7 | PS 38:7 |  |  |  |  |  |  |  |  |  |  |  |
| 780.5536 | 18.0 | PC 36:5 |  |  |  |  |  |  |  |  |  |  |  |
| 304.2843 | 11.7 | Phytosphingolipid |  |  |  |  |  |  |  |  |  |  |  |
| 732.4805 | 11.7 | PS(18:2(9Z,12Z)/14:0) |  |  |  |  |  |  |  |  |  |  |  |
| 796.6212 | 19.3 | PC ether 38:4 |  |  |  |  |  |  |  |  |  |  |  |
| 635.5024 | 13.1 | SM PE 32:0 |  |  |  |  |  |  |  |  |  |  |  |
| 647.5129 | 13.4 | SM(d18:1/12:0) |  |  |  |  |  |  |  |  |  |  |  |
| 768.5544 | 12.0 | PE 38:4 |  |  |  |  |  |  |  |  |  |  |  |
| 768.1225 | 7.3 | CoA |  |  |  |  |  |  |  |  |  |  |  |
| 890.6561 | 3.4 | GBC 36:1 |  |  |  |  |  |  |  |  |  |  |  |
| 702.5894 | 3.0 | GC 34:0 |  |  |  |  |  |  |  |  |  |  |  |
| 591.4409 | 2.8 | Coenzyme Q6 |  |  |  |  |  |  |  |  |  |  |  |
| 318.3001 | 12.4 | 4-hydroxysphinganine |  |  |  |  |  |  |  |  |  |  |  |
| 724.5273 | 12.0 | PE ether 36:4 |  |  |  |  |  |  |  |  |  |  |  |
| 756.481 | 11.7 | PS 34:4 |  |  |  |  |  |  |  |  |  |  |  |
| 742.583 | 3.1 | GC 36:2 hydroxy |  |  |  |  |  |  |  |  |  |  |  |
| 702.5071 | 18.2 | PC 30:2 |  |  |  |  |  |  |  |  |  |  |  |
| 678.5441 | 12.4 | PE ether 32:1 |  |  |  |  |  |  |  |  |  |  |  |
| 580.3972 | 12.6 | PE 24:0 |  |  |  |  |  |  |  |  |  |  |  |
| 786.6363 | 12.2 | PE ether 40:1 |  |  |  |  |  |  |  |  |  |  |  |
| 674.5115 | 12.2 | PE ether 32:1 |  |  |  |  |  |  |  |  |  |  |  |
| 784.6213 | 12.0 | PE ether 40:2 |  |  |  |  |  |  |  |  |  |  |  |
| 734.4966 | 3.3 | PS(18:1(9Z)/14:0) |  |  |  |  |  |  |  |  |  |  |  |
| 892.7391 | 2.8 | TG 56:7 |  |  |  |  |  |  |  |  |  |  |  |
| 794.6048 | 19.3 | PC ether 38:5 |  |  |  |  |  |  |  |  |  |  |  |
| 732.5534 | 18.1 | PC 32:1 |  |  |  |  |  |  |  |  |  |  |  |
| 287.2211 | 3.0 | Hexadecanedioic acid |  |  |  |  |  |  |  |  |  |  |  |
| 946.7177 | 2.8 | GBC 40:1 |  |  |  |  |  |  |  |  |  |  |  |
| 760.5848 | 18.1 | PC 34:1 |  |  |  |  |  |  |  |  |  |  |  |
| 304.2841 | 10.4 | Phytosphingolipid |  |  |  |  |  |  |  |  |  |  |  |
| 512.5044 | 2.8 | SP 32:0 |  |  |  |  |  |  |  |  |  |  |  |
| 722.5106 | 12.0 | PE ether 36:5 |  |  |  |  |  |  |  |  |  |  |  |
| 301.216 | 3.0 | Retinoic acid |  |  |  |  |  |  |  |  |  |  |  |
| 728.5226 | 3.3 | PE(20:3(8Z,11Z,14Z)/15:0) |  |  |  |  |  |  |  |  |  |  |  |
| 782.6058 | 11.9 | PE ether 40:3 |  |  |  |  |  |  |  |  |  |  |  |
| 798.6006 | 12.0 | PE 40:3 |  |  |  |  |  |  |  |  |  |  |  |
| 756.5535 | 17.9 | PC 34:3 |  |  |  |  |  |  |  |  |  |  |  |
| 780.5897 | 11.9 | PE ether 40:4 |  |  |  |  |  |  |  |  |  |  |  |
| 760.5848 | 16.4 | PS 34:2 |  |  |  |  |  |  |  |  |  |  |  |
| 620.5972 | 2.9 | Cer 40:2 |  |  |  |  |  |  |  |  |  |  |  |
| 754.537 | 18.0 | PC 34:4 |  |  |  |  |  |  |  |  |  |  |  |
| 800.6165 | 12.0 | PE 40:2 |  |  |  |  |  |  |  |  |  |  |  |
| 728.5601 | 19.5 | PE(P-18:1(9Z)/18:1(9Z)) |  |  |  |  |  |  |  |  |  |  |  |
| 802.632 | 12.1 | PE 40:1 |  |  |  |  |  |  |  |  |  |  |  |
| 756.5537 | 16.2 | PC 34:3 |  |  |  |  |  |  |  |  |  |  |  |
| 730.5383 | 16.3 | PC 32:2 |  |  |  |  |  |  |  |  |  |  |  |
| 786.6007 | 17.9 | PC 36:2 |  |  |  |  |  |  |  |  |  |  |  |
| 652.6597 | 3.3 | SP 42:0 |  |  |  |  |  |  |  |  |  |  |  |
| 730.538 | 18.0 | PC 32:2 |  |  |  |  |  |  |  |  |  |  |  |
| 300.2893 | 5.7 | Sphingosine |  |  |  |  |  |  |  |  |  |  |  |
| 704.5224 | 18.2 | PC 30:1 |  |  |  |  |  |  |  |  |  |  |  |
| 780.5526 | 15.4 | PC 36:5 |  |  |  |  |  |  |  |  |  |  |  |
| 728.5229 | 18.0 | PE(20:3(8Z,11Z,14Z)/15:0) |  |  |  |  |  |  |  |  |  |  |  |
| 828.6479 | 12.0 | PE 42:2 |  |  |  |  |  |  |  |  |  |  |  |
| 524.3707 | 18.5 | Lyso PC 18:0 |  |  |  |  |  |  |  |  |  |  |  |
