# Supplementary material for: Mapping an Atlas of Tissue-Specific Drosophila melanogaster Metabolomes by High Resolution Mass Spectrometry
Source: PLoS One. 2013 Oct 29;8(10):e78066. doi: 10.1371/journal.pone.0078066 (PMC3812166; doi:10.1371/journal.pone.0078066)
Supplement: Table S5 — Putatively identified negatively charged lipids in Drosophila tissues ranked according to abundance in the whole fly. Mass deviation from the exact masses in the database were <2 ppm. (DOCX) [file pone.0078066.s005.docx]

**Table S5** Putatively identified negatively charged lipids in Drosophila tissues ranked according to abundance in the whole fly. Mass deviation from the exact masses in the database were < 2ppm.

| MZ | RT | Name | WF | Crop | MG | HD | AT | PT | HG | OV | TEST | ACCG | CUT |
| --- | --- | --- | --- | --- | --- | --- | --- | --- | --- | --- | --- | --- | --- |
| 595.2886 | 6.7 | Lyso PI 18:1 |  |  |  |  |  |  |  |  |  |  |  |
| 569.2732 | 6.7 | Lyso PI 16:0 |  |  |  |  |  |  |  |  |  |  |  |
| 593.2733 | 6.7 | Lyso PI 18:2 |  |  |  |  |  |  |  |  |  |  |  |
| 861.5493 | 5.0 | PI 36:2 |  |  |  |  |  |  |  |  |  |  |  |
| 745.5016 | 3.3 | GPG 34:2 |  |  |  |  |  |  |  |  |  |  |  |
| 719.4864 | 4.9 | GPG 32:1 |  |  |  |  |  |  |  |  |  |  |  |
| 745.5017 | 4.8 | GPG 34:2 |  |  |  |  |  |  |  |  |  |  |  |
| 743.486 | 3.3 | GPG 34:3 |  |  |  |  |  |  |  |  |  |  |  |
| 765.4703 | 3.3 | GPG 36:6 |  |  |  |  |  |  |  |  |  |  |  |
| 805.4859 | 3.4 | PI 32:2 |  |  |  |  |  |  |  |  |  |  |  |
| 857.5175 | 3.3 | PI 36:4 |  |  |  |  |  |  |  |  |  |  |  |
| 747.5176 | 3.3 | GPG 34:1 |  |  |  |  |  |  |  |  |  |  |  |
| 805.4858 | 3.2 | PI 32:2 |  |  |  |  |  |  |  |  |  |  |  |
| 773.5327 | 3.3 | PG 36:2 |  |  |  |  |  |  |  |  |  |  |  |
| 859.5334 | 5.0 | PI 36:3 |  |  |  |  |  |  |  |  |  |  |  |
| 719.4865 | 3.3 | GPG 32:1 |  |  |  |  |  |  |  |  |  |  |  |
| 769.5012 | 3.3 | GPG 36:4 |  |  |  |  |  |  |  |  |  |  |  |
| 859.5325 | 4.9 | PI 36:3 |  |  |  |  |  |  |  |  |  |  |  |
| 859.5329 | 3.3 | PI 36:3 |  |  |  |  |  |  |  |  |  |  |  |
| 741.4706 | 3.3 | GPG 34:4 |  |  |  |  |  |  |  |  |  |  |  |
| 833.5164 | 3.4 | PI 34:2 |  |  |  |  |  |  |  |  |  |  |  |
| 861.5482 | 3.3 | PI 36:2 |  |  |  |  |  |  |  |  |  |  |  |
| 743.4862 | 4.8 | GPG 34:3 |  |  |  |  |  |  |  |  |  |  |  |
| 833.5174 | 5.2 | PI 34:2 |  |  |  |  |  |  |  |  |  |  |  |
| 833.5164 | 3.1 | PI 34:2 |  |  |  |  |  |  |  |  |  |  |  |
| 721.5017 | 3.3 | GPG 32:0 |  |  |  |  |  |  |  |  |  |  |  |
| 767.4858 | 3.3 | GPG 36:5 |  |  |  |  |  |  |  |  |  |  |  |
| 857.5173 | 5.2 | PI 36:4 |  |  |  |  |  |  |  |  |  |  |  |
| 747.5179 | 4.8 | GPG 34:1 |  |  |  |  |  |  |  |  |  |  |  |
| 717.4709 | 3.3 | GPG 32:2 |  |  |  |  |  |  |  |  |  |  |  |
| 741.4706 | 4.8 | GPG 34:4 |  |  |  |  |  |  |  |  |  |  |  |
| 439.3795 | 3.3 | MG 24:1 |  |  |  |  |  |  |  |  |  |  |  |
| 963.5028 | 3.3 | PIP 38:5 |  |  |  |  |  |  |  |  |  |  |  |
| 807.5013 | 5.2 | PI 32:1 |  |  |  |  |  |  |  |  |  |  |  |
| 597.3048 | 6.7 | Lyso PI 18:0 |  |  |  |  |  |  |  |  |  |  |  |
| 767.4858 | 4.7 | GPG 36:5 |  |  |  |  |  |  |  |  |  |  |  |
| 887.5637 | 5.0 | PI 38:3 |  |  |  |  |  |  |  |  |  |  |  |
| 807.5021 | 3.4 | PI 32:1 |  |  |  |  |  |  |  |  |  |  |  |
| 883.533 | 6.3 | PI 38:5 |  |  |  |  |  |  |  |  |  |  |  |
| 661.5191 | 13.3 | PA(16:0e/18:0) |  |  |  |  |  |  |  |  |  |  |  |
| 327.254 | 3.3 | MG 16:1 |  |  |  |  |  |  |  |  |  |  |  |
| 509.2882 | 4.3 | Lyso GPG 18:1 |  |  |  |  |  |  |  |  |  |  |  |
| 771.517 | 3.2 | GPG 36:3 |  |  |  |  |  |  |  |  |  |  |  |
| 509.2883 | 6.9 | Lyso GPG 18:1 |  |  |  |  |  |  |  |  |  |  |  |
| 717.4709 | 4.9 | GPG 32:2 |  |  |  |  |  |  |  |  |  |  |  |
| 885.5491 | 3.4 | PI 38:4 |  |  |  |  |  |  |  |  |  |  |  |
| 509.2886 | 5.6 | Lyso GPG 18:1 |  |  |  |  |  |  |  |  |  |  |  |
| 827.7117 | 3.1 | [GL (16:0/17:0/17:0)] 1-(9Z-hexadecenoyl)-2,3-di-(9Z-heptadecenoyl)-sn-glycerol |  |  |  |  |  |  |  |  |  |  |  |
| 483.273 | 5.9 | Lyso GPG 16:0 |  |  |  |  |  |  |  |  |  |  |  |
| 889.5804 | 3.4 | PI 38:2 |  |  |  |  |  |  |  |  |  |  |  |
| 765.4701 | 4.7 | GPG 36:6 |  |  |  |  |  |  |  |  |  |  |  |
| 775.5491 | 4.8 | GPG 36:1 |  |  |  |  |  |  |  |  |  |  |  |
| 773.533 | 6.1 | PG 36:2 |  |  |  |  |  |  |  |  |  |  |  |
| 721.5035 | 4.9 | GPG 32:0 |  |  |  |  |  |  |  |  |  |  |  |
| 703.5272 | 2.7 | GP 36:0 |  |  |  |  |  |  |  |  |  |  |  |
| 885.5488 | 3.4 | PI 38:4 |  |  |  |  |  |  |  |  |  |  |  |
| 835.5328 | 6.5 | PI 34:1 |  |  |  |  |  |  |  |  |  |  |  |
| 329.2695 | 3.3 | MG 16:0 |  |  |  |  |  |  |  |  |  |  |  |
| 659.4663 | 3.3 | [GL (20:5/20:5)] 1,2-di-(5Z,8Z,11Z,14Z,17Z-eicosapentaenoyl)-sn-glycerol |  |  |  |  |  |  |  |  |  |  |  |
| 823.6806 | 3.4 | [GL (16:0/17:2/17:2)] 1-(9Z-hexadecenoyl)-2,3-di-(9Z,12Z-heptadecadienoyl)-sn-glycerol |  |  |  |  |  |  |  |  |  |  |  |
| 861.548 | 6.3 | PI 36:2 |  |  |  |  |  |  |  |  |  |  |  |
| 673.4815 | 11.2 | PA(16:0/18:1(9Z)) |  |  |  |  |  |  |  |  |  |  |  |
